# Hippocampal subfield differences in people with and without recreational ketamine use: Insights from multi‐modal neuroimaging

**DOI:** 10.1111/add.70331

**Published:** 2026-01-29

**Authors:** Yi‐Hsuan Liu, Chia‐Chun Hung, Marc N. Potenza, Kun‐Hsien Chou, Pei‐Lin Lee, Chu‐Chung Huang, Chiang‐Shan R. Li, Tony Szu‐Hsien Lee, Ching‐Po Lin

**Affiliations:** ^1^ Institute of Neuroscience National Yang Ming Chiao Tung University Taipei Taiwan; ^2^ Department of Neurology Columbia University New York NY USA; ^3^ Center for Addiction Prevention and Policy Research National Taiwan Normal University Taipei Taiwan; ^4^ Continuing Education Master's Program of Addiction Prevention and Treatment National Taiwan Normal University Taipei Taiwan; ^5^ Department of Psychiatry, School of Medicine Yale University New Haven CT USA; ^6^ The Child Study Center, School of Medicine Yale University New Haven CT USA; ^7^ Department of Neuroscience and the Wu Tsai Institute Yale University New Haven CT USA; ^8^ The Connecticut Council on Problem Gambling Wethersfield CT USA; ^9^ The Connecticut Mental Health Center New Haven CT USA; ^10^ Brain Research Center National Yang Ming Chiao Tung University Taipei Taiwan; ^11^ Center for Healthy Longevity and Aging Sciences National Yang Ming Chiao Tung University Taipei Taiwan; ^12^ School of Psychology and Cognitive Science East China Normal University Shanghai China; ^13^ Department of Health Promotion and Health Education National Taiwan Normal University Taipei Taiwan; ^14^ Department of Education and Research Taipei City Hospital Taipei Taiwan; ^15^ Institute of Biomedical Engineering and Nanomedicine, National Health Research Institutes Miaoli Taiwan

**Keywords:** functional neuroimaging, hippocampus, ketamine, magnetic resonance imaging, N‐methyl‐D‐aspartate receptors, recreational substance use, substance‐related disorders, working memory

## Abstract

**Background and aims:**

Recreational ketamine use has increased globally and is associated with psychiatric and cognitive concerns. The hippocampus in preclinical models shows damage and working‐memory disruption with repeated dosing. However, whether specific hippocampal subregions may differ in people with chronic ketamine use remains unclear. In Taiwan, ketamine is predominantly consumed by smoking ketamine mixed with tobacco, producing smoking‐related behavioral profiles like non‐ketamine tobacco use participants (TUs). We therefore examined individuals with urine‐confirmed ketamine as the only detected substance who reported predominantly smoking‐administered recreational use (KUs) and used TUs as controls. This study aimed to: (1) characterize ketamine‐use patterns and psychiatric symptoms; (2) compare working‐memory and affective‐behavioral measures between KUs and TUs; (3) quantify group differences in hippocampal subregion volumes; and (4) assess group differences in functional connectivity (FC) of identified subregions and relationships with neurotransmitter receptor distributions.

**Design:**

Cross‐sectional case‐control study with cognitive testing and neuroimaging.

**Setting:**

Community‐based recruitment in Taiwan.

**Participants:**

58 KUs (44 males; mean age = 21.00 ± 4.57) and 73 TUs (52 males; mean age = 24.34 ± 5.86).

**Measurements:**

Ketamine‐use patterns (Addiction Severity Index), psychiatric symptoms [Symptom Checklist‐90‐Revised (SCL‐90‐R)], working‐memory (N‐back), affective‐behavioral measures [Barratt Impulsiveness Scale (BIS‐11), Buss and Perry Aggression Questionnaire (BPAQ), Sensitivity to Punishment and Sensitivity to Reward Questionnaire (SPSRQ)], hippocampal subfield volumes (FreeSurfer) and functional connectivity (FC) of identified subregions (seed‐based analysis). Spatial correspondence with N‐methyl‐D‐aspartate (NMDA) receptor density was evaluated using JuSpace.

**Findings:**

Heavier ketamine use was associated with greater psychological distress [Global Severity Index (GSI) r = 0.343, *P* = 0.011], particularly anxiety (r = 0.457, *P* < 0.001) and hostility (r = 0.442, *P* < 0.001). Although self‐reported impulsivity, aggression and reward/punishment sensitivity did not differ between groups, KUs showed reduced accuracy under higher working‐memory load [2‐back: F(1, 124) = 4.16, *P* = 0.04, partial *η* ^2^ = 0.03; 1‐back: F(1, 124) = 8.10, *P* = 0.005, η2 = 0.06]. KUs displayed reduced left hippocampal volume [F(1, 119) = 4.23, *P* = 0.04, η2 = 0.03], most marked in the hippocampal‐amygdaloid‐transition‐area [HATA; F(1, 119) = 10.52, *P* = 0.002, η2 = 0.08]. KUs also showed increased FC between left HATA and frontal, cingulate, temporal, subcortical, insular and cerebellar regions (*P* < 0.05, AlphaSim corrected), which correlated with NMDA‐receptor distributions (z = 0.30, *P* = 0.005, false discovery rate corrected).

**Conclusions:**

Recreational smoking‐administered ketamine use appears to be associated with dose‐dependent psychiatric symptoms, load‐dependent working memory impairment, selective hippocampal subregion volumetric differences and altered network connectivity aligned with N‐methyl‐D‐aspartate‐ (NMDA) receptor distribution.

## INTRODUCTION

Ketamine has gained increasing clinical attention because of its rapid antidepressant effects, including in people with treatment‐resistant depression [1, 2]. Concurrently, the increasing prevalence of non‐medical/recreational ketamine use has raised significant public health concerns. Although ketamine is considered safe within controlled clinical settings, it possesses potential abuse liability [3, 4, 5]. Recent epidemiological reports document rises in ketamine misuse and ketamine use disorder globally, including increased illicit ketamine distribution in the United States and a doubling of treatment admissions in the United Kingdom between 2019 and 2023 [3, 6, 7]. At doses similar to those used therapeutically, ketamine can produce reinforcing effects via N‐methyl‐D‐aspartate (NMDA) receptor antagonism and downstream dopaminergic activity within reward circuits [8, 9]. Chronic recreational misuse has been associated with cognitive impairments, dissociation, affective disturbances and other psychiatric symptoms [10, 11, 12, 13, 14], suggesting a need to clarify concerns related to repeated exposure.

The hippocampus expresses high levels of NMDA receptors and has been implicated in learning, memory and affect regulation [15, 16]. Long‐term recreational ketamine use has been associated with impaired spatial memory performance and reduced hippocampal activation during spatial tasks [17, 18]. In rats, hippocampal damage and working‐memory disruption has been observed at anesthetic doses of 40 mg/kg or 75 mg/kg [17, 18]. By contrast, therapeutic ketamine can enhance hippocampal plasticity through BDNF (brain‐derived neurotrophic factor)/AKT (the protein kinase B)/mTOR (mammalian target of rapamycin) signaling [19] and has been associated with acute increases in hippocampal subregion volume [20]. These seemingly opposing findings underscore the importance of understanding differences related to short‐term therapeutic administration and chronic recreational use. Functional connectivity (FC) studies further suggest that ketamine may produce distinct whole‐brain connectivity patterns with substantial individual variability [21], yet how these patterns relate specifically to hippocampal structure in relation to recreational use remains unclear.

Previously, we associated long‐term recreational ketamine use with smaller gray matter volumes across multiple cortical regions, particularly the precuneus, insula, inferior parietal lobule, dorsolateral prefrontal cortex and medial orbitofrontal cortex [22]. Notably, individuals who initiated ketamine use during adolescence exhibited more pronounced gray matter reductions in the precuneus, accompanied by disrupted default mode network FC, suggesting potentially heightened neural vulnerability during early neurodevelopmental periods. However, direct links between these structural and connectivity findings and cognitive measures remain unclear. In a subsequent study, we also observed that individuals with ketamine use demonstrated poorer short‐term working memory performance and elevated impulsivity compared with participants with tobacco use and without ketamine use (TUs) [23]. These behavioral findings suggest potential disruptions in cognitive control and reward processing, yet the underlying neurobiological mechanisms have not been fully delineated.

However, these prior studies did not examine hippocampal subregions. Given its high density of NMDA receptors and integrative role in memory and behavioral dysregulation in addictions, it remains unclear whether specific hippocampal subregions may show selective vulnerability to chronic ketamine exposure and whether such structural measures relate to FC differences or behavioral tendencies. Furthermore, many prior human studies are confounded by polysubstance use or involve low‐dose, short‐term exposure in clinical or experimental contexts that differ substantially from real‐world recreational patterns. To address these limitations, the present study examined individuals with urine–toxicology‐supported ketamine as the only substance detected at the time of testing, predominantly smoking‐administered recreational ketamine use (KUs). Recreational ketamine use in Taiwan is often consumed through smoking joints, as evidenced by our previous study in which 94.6% (*n* = 175) of KUs reported this method [23]. Therefore, TUs were selected as a comparison group to control for lifestyle and substance‐use behavioral profiles, while avoiding neurobiological confounds associated with tobacco use. Accordingly, the present study had four aims. First, we characterized patterns of recreational ketamine use and examined their associations with psychiatric symptom profiles. Second, we compared the behavioral phenotype of KUs and TUs, including working memory performance and affective‐behavioral tendencies (impulsivity, aggression and sensitivity to reward/punishment). Third, we quantified the differences in hippocampal subregion volumes between the two groups using high‐resolution structural magnetic resonance imaging (MRI). Fourth, we assessed group differences in FC within identified hippocampal subregions and evaluated whether FC measures aligned with NMDA receptor distribution patterns. Together, these aims allow us to determine whether chronic recreational ketamine use relates to selective hippocampal subregion measures and corresponding network‐level and behavioral measures, thereby addressing a critical gap in knowledge.

## METHODS

### Study design

This study used a cross‐sectional case–control design comparing KUs to TUs. KUs reported ongoing non‐medical ketamine use, predominantly via smoking ketamine mixed with tobacco. TUs were selected to match lifestyle characteristics and nicotine exposure while excluding potential influences of illicit substances. All participants completed structured clinical interviews, urine toxicology screening, standardized neuropsychological and behavioral assessments and high‐resolution MRI scanning. Age, sex, years of education and nicotine dependence were included as covariates in subsequent analyses.

### Participants and clinical assessments

Because recreational ketamine use is illegal and difficult to research in Taiwan, participants (age, 18–40 years) were recruited using respondent‐driven sampling [24, 25] from community locations in northern Taiwan within approximately 40 km of the research center. Following an initial telephone screening, eligible individuals completed in‐person clinical interviews and urine toxicology testing.

Participants were classified into two groups: KUs and TUs. KUs reported ongoing non‐medical ketamine use and tested positive for ketamine, but negative for other illicit substances (e.g. amphetamines, cannabis and 3,4‐methylenedioxymethamphetamine). TUs reported ongoing tobacco use and tested negative for all illicit substances. Eight participants were excluded because of MRI contraindications, excessive head motion or scanning discomfort, resulting in a final sample of 58 KUs and 73 TUs. All participants provided written informed consent, and the study was approved by the National Taiwan Normal University Research Ethics Committee.

Urine toxicology testing was conducted within 2 hours before MRI scanning using the Firstep Multi‐Drug Test (https://www.firstep.com.tw/en-us/Product-Detail/D014), which detects recent drug exposure within 1 to 2 days. Substance use characteristics were assessed using a modified Addiction Severity Index (ASI) [26], which included ketamine‐specific items (route of administration, estimated dose per use, frequency and total use over the past 30 days, duration of use and age at onset). Ketamine‐related urinary symptoms were also recorded. Tobacco dependence severity was assessed using the Fagerström Test for Nicotine Dependence (FTND) [27], along with additional smoking history (age of initiation, years of smoking, smoking frequency and maximum daily consumption).

### Neuropsychological and neurobehavioral testing

Current psychological symptoms were assessed using the Symptom Checklist‐90‐Revised (SCL‐90‐R), which measures psychiatric symptomatology across nine domains. The Global Severity Index (GSI) was used as an overall indicator of psychological distress [28, 29]. Impulsivity was assessed using the Barratt Impulsiveness Scale (BIS‐11), a 30‐item self‐report questionnaire yielding a total impulsivity score [30]. Aggression was measured using the Buss–Perry Aggression Questionnaire (BPAQ), which provides a total aggression score derived from physical aggression, verbal aggression, anger and hostility subscales [31]. Sensitivity to reward and punishment was evaluated using the Sensitivity to Punishment and Sensitivity to Reward Questionnaire (SPSRQ), generating separate sensitivity‐to‐punishment and sensitivity‐to‐reward scores [32].

Working memory was assessed using a sequential‐letter N‐back task [33] consisting of three load levels (0‐back, 1‐back and 2‐back). The 0‐back condition required responses to a target letter, whereas the 1‐back and 2‐back conditions required matching to the letter presented one or two trials prior. Accuracy and reaction time (RT) were recorded for each condition. Detailed task procedures are described in the Methods Section S1.

### MRI data acquisition protocol

MRI data were acquired using standard T1 and resting‐state function MRI (fMRI) sequences. Detailed acquisition parameters are provided in Methods Section S2.

### Structural MRI data processing and quality control

Structural MRI data were processed using the FreeSurfer automated cortical reconstruction pipeline (FreeSurfer version 7.1.1; https://surfer.nmr.mgh.harvard.edu/fswiki/FreeSurferAnalysisPipelineOverview) [34]. Standard pre‐processing included skull stripping, intensity normalization, subcortical segmentation and cortical surface reconstruction [35, 36, 37, 38, 39, 40, 41, 42]. Total intracranial volume (TIV) was computed automatically. Hippocampal subfield segmentation was performed using FreeSurfer's probabilistic atlas‐based segmentation procedure derived from ultra–high‐resolution ex vivo MRI data [43]. Bilateral volumes were extracted for the parasubiculum, presubiculum (head and body), subiculum, CA1, CA3, CA4, granule cell and molecular layer of the dentate gyrus, molecular layer, hippocampal–amygdaloid transition area (HATA), fimbria, tail and hippocampal fissure.

All hippocampal segmentations underwent visual quality control by a trained rater experienced in FreeSurfer hippocampal subfield inspection. Scans exhibiting motion artifacts, suboptimal skull stripping or inaccurate subfield boundaries were reprocessed or excluded. Only datasets that passed this quality control procedure were included in the final analyses.

### Resting‐state fMRI pre‐processing and quality control

Resting‐state fMRI pre‐processing was performed using Analysis of Functional NeuroImages (AFNI) (v22.1.01), FMRIB Software Library (FSL) (v6.0.5.1), Advanced Normalization Tools (ANTs) (v2.3.5) and SPM12. The first 10 volumes were discarded, and images were corrected for slice timing and head motion (MCFLIRT) [44], followed by skull stripping (FSL Brain Extraction Tool, BET) [45] and spatial normalization to each participant's T1 image and then to Montreal Neurological Institute (MNI) space using ANTs SyN (Advanced Normalization Tools Symmetric Normalization) registration [46]. Nuisance regression included the Friston 24‐parameter motion model [47], mean white matter and cerebrospinal fluid signals and polynomial trends, followed by temporal band‐pass filtering (0.008–0.08 Hz) and spatial smoothing (6 mm full width at half maximum).

Participants with >2 mm translation, >2° rotation, mean frame‐wise displacement (FD) >0.2 mm, or <5 minutes of low‐motion data (FD <0.2 mm) were excluded [48, 49]. To ensure reliable signal within hippocampal subfields, temporal signal‐to‐noise ratio (tSNR) was computed and participants with tSNR <50 were removed [50, 51]. After quality control, 24 KUs and 33 TUs were retained for FC analyses.

Seed‐based connectivity analysis was performed using the hippocampal subfield that showed significant volumetric group differences. The mean time series from the seed was correlated with all brain voxels, and correlation values were converted to Fisher z‐scores for group‐level analysis.

### Associations between hippocampal seed‐based functional connectivity and neurochemical maps

To test whether ketamine‐related structural and FC findings were potentially associated with neurochemical architecture, we conducted receptor‐mapping analyses using JuSpace (version 1.5) [52]. This framework enables quantitative spatial correlation between seed‐to‐voxel FC difference maps and high‐resolution positron emission tomography (PET)/single photon emission computed tomography (SPECT)‐derived neurotransmitter receptor and transporter templates.

Given ketamine's primary action as an NMDA receptor antagonist and its downstream effects on multiple neuromodulatory systems, 17 neurochemical maps were included in the analysis: serotonergic receptors (5‐HT1A, 5‐HT1B, 5‐HT2A, 5‐HT4), dopaminergic measures [D1 receptors, D2 receptors, ^18^F‐fluorodopa (FDOPA), Dopamine transporter (DAT)], glutamatergic receptors (NMDA, mGluR5), GABA_A receptors, cholinergic vesicular transporter (VAChT), noradrenergic transporter (NAT), mu‐opioid receptors (MOR), CB1 cannabinoid receptor, serotonin transporter (SERT) and cerebral blood flow (CBF).

Spatial correlations were computed between the KU–TU FC contrast map (Fisher z‐transformed) and each neurochemical template using voxel‐wise Pearson correlation. Statistical significance was assessed using permutation‐based testing (10 000 permutations) to generate empirical null distributions. Multiple comparisons across the 17 neurotransmitter maps were controlled using false discovery rate (FDR) correction (q < 0.05).

### Statistical analysis

#### Sample size estimation

An *a priori* power analysis using G*Power 3.1 [53] was performed based on an effect size (Cohen's *d* = 0.79) from prior structural findings in people with chronic ketamine use [54], yielding a required sample size of 43 participants per group with α = 0.05 and power = 0.95.

#### Demographic and clinical characteristics

All statistical analyses for demographic and clinical variables were performed in IBM SPSS Statistics version 29 (IBM). Continuous variables were compared between groups using independent‐samples *t* tests, and categorical variables were examined using χ^2^ tests. Neuropsychological and behavioral measures were analyzed using analysis of covariance (ANCOVA), adjusting for age, sex, years of education and nicotine dependence (FTND score). Statistical significance was set at *P* < 0.05.

#### Associations between ketamine use and psychiatric symptoms

Within KUs, partial Pearson correlations were performed to examine associations between ketamine use characteristics (average dose, total use in the past 30 days, frequency of use and duration of use) and both the GSI and SCL‐90‐R domain scores, controlling for age, sex, years of education and FTND score. Bonferroni correction (adjusted *P* < 0.05) was applied across the nine SCL‐90‐R domains.

#### Group differences in hippocampal volume and subfields

Group differences in bilateral hippocampal volume and hippocampal subfield volumes were tested using ANCOVAs controlling for age, sex, years of education, FTND and TIV. Bonferroni correction was used for multiple comparisons (α = 0.025 for hemispheres; α = 0.002 for 24 subfields).

#### Hippocampal subfield‐based functional connectivity

Voxel‐wise comparisons of seed‐based functional connectivity were conducted in SPM12 using general linear models with the same covariates. Cluster‐level correction used AFNI's 3dClustSim (10 000 iterations) using an explicit gray‐matter mask, applying a combined threshold of voxel‐wise *P* < 0.05 and cluster extent ≥462 voxels, corresponding to a family‐wise error rate of α = 0.05 (AFNI version 22.1.13; https://afni.nimh.nih.gov/).

#### *Post hoc* sensitivity analyses

To evaluate the robustness of group differences in hippocampal structure, *post hoc* sensitivity analyses were conducted. First, propensity‐score matching was performed using nearest‐neighbor matching with caliper widths of 0.4 and 0.2 on age, sex, years of education and FTND scores, and ANCOVAs were repeated in the matched subgroups to confirm whether the primary effects remained. To account for psychiatric symptom severity, GSI scores were added as a covariate, and group × GSI, group × sex and group × FTND interaction terms were tested to assess potential moderating effects. Additional models further controlled for nicotine‐related factors (daily cigarette consumption and age of smoking onset) and substance use severity (ASI drug and alcohol composite scores). Within the KU group, partial correlations were conducted to examine associations between hippocampal volume and patterns of ketamine use (frequency and duration), adjusting for age, sex, years of education, FTND and GSI. Only effects that remained statistically significant under the corrected thresholds are reported.

### Pre‐registration

The primary research question and analysis plan were not pre‐registered on a publicly available platform. Therefore, the analyses and results should be considered exploratory.

## RESULTS

### Participants and substance use characteristics

All KUs met Diagnostic and Statistical Manual of Mental Disorders, fifth edition criteria for at least mild substance use disorder (2–3 symptoms) and reported ongoing recreational ketamine use. Sample characteristics and substance use characteristics are summarized in Table 1. The predominant route of ketamine administration was smoking ketamine mixed with tobacco, and four KUs additionally reported intranasal use. Six KUs reported urinary frequency, and two had previously sought urological consultation. Within KUs, longer duration of ketamine use was positively correlated with higher dose per use (r = 0.331, *P* = 0.014) and greater total monthly ketamine use (r = 0.297, *P* = 0.029). Comparisons of tobacco use severity and ASI scores between groups are reported in Results Section S1.

**TABLE 1:** Demographics.

Mean (SD)	Ketamine use	Tobacco use	Statistic
No.	58	73	0.000
Age (in years)	21 (4.57)	24.34 (5.86)	*t*_129_ = −3.56, *P* < 0.001***, Cohen's *d* = −0.63, 95% CI = −0.98 to −0.27
Sex (male: female)	44:14	52:21	χ^2^ (1) = 0.354, *P* = 0.552
Education (years)	9.57 (2.26)	12.08 (2.05)	*t*_129_ = −6.66, *P* < 0.001***, Cohen's *d* = −1.17, 95% CI = −1.54 to −0.80
Ketamine use information			
Last 30 days frequency of ketamine use	11.34 (9.86)	N/A	N/A
Dose of ketamine use at one time (in g)	1.141 (1.63)	N/A	N/A
Total amount of ketamine used in the last 30 days (in g)	14.66 (21.23)	N/A	N/A
Estimated lifetime duration since first exposure to ketamine (in months)	41.25 (38.52)	N/A	N/A
Age of ketamine use onset	17.56 (2.85)	N/A	N/A
Tobacco use information			
Fagerstrom test for nicotine dependence	5.18 (2.39)	3.78 (2.42)	*t*_129_ = 3.32, *P* = 0.001***, Cohen's *d* = 0.59
Estimated lifetime duration since first exposure to tobacco use (in years)	5.37 (4.65)	6.47 (5.51)	*t*_129_ = −1.21, *P* = 0.227, Cohen's *d* = −0.21
Daily cigarette consumption	19.62 (10.17)	13.22 (8.33)	*t*_129_ = 3.87, *P* < 0.001***, Cohen's *d* = 0.70
Age of tobacco use onset	15.63 (2.40)	17.87 (4.41)	*t*_129_ = −3.48, *P* < 0.001***, Cohen's *d* = −0.61
Tobacco use frequency (in days/week)	6.16 (2.07)	5.69 (2.24)	*t*_129_ = 1.24, *P* = 0.219, Cohen's *d* = 0.22
Addiction Severity Index			
Alcohol composite score	0.12 (0.13)	0.12 (0.10)	*t*_129_ = 0.02, *P* < 0.981, Cohen's *d* = 0.0004
Drug composite score	0.06 (0.08)	0.006 (0.02)	*t*_129_ = 6.00, *P* < 0.001***, Cohen's *d* = 1.06
Neuropsychological and neurobehavioral testing^a^			
Symptom Checklist‐90	141.24 (56.00)	142.05 (59.35)	*F*_1, 125_ = 0.01, *P* = 0.91, partial *η* ^2^ = 0.00, 95% CI = −21.54 to 24.28
Barratt Impulsiveness Scale‐11	73.29 (8.13)	69.23 (8.76)	*F*_1, 125_ = 1.44, *P* = 0.23, partial *η* ^2^ = 0.01, 95% CI = −1.33 to 5.44
Buss‐Perry Aggression Questionnaire	69.79 (22.55)	70.76 (20.13)	*F*_1, 125_ = 1.60, *P* = 0.21, partial *η* ^2^ = 0.01, 95% CI = −14.17 to 3.12
Sensitivity to Punishment and Sensitivity to Reward Questionnaire	23.04 (11.38)	24.14 (7.28)	*F*_1, 125_ = 1.96, *P* = 0.16, partial *η* ^2^ = 0.02, 95% CI = −7.26 to 1.24
N‐back working memory task performance^a^			
Accuracy at 0‐back level	0.86 (0.21)	0.95 (0.15)	*F*_**1, 124**_ = 1.72., *P* = 0.19, partial *η* ^2^ = 0.01
Accuracy at 1‐back level	0.77 (0.20)	0.91 (0.13)	*F*_**1, 124**_ = 8.10., *P* = 0.005**, partial *η* ^2^ = 0.06
Accuracy at 2‐back level	0.63 (0.24)	0.76 (0.19)	*F*_**1, 124**_ = 4.16., *P* = 0.04*, partial *η* ^2^ = 0.03
RTs at 0‐back level	477.98 (86.55)	449.64 (69.22)	*F*_**1, 124**_ = 0.60., *P* = 0.44, partial *η* ^2^ = 0.005
RTs at 1‐back level	551.96 (102.75)	497.92 (92.72)	*F*_**1, 124**_ = 3.86., *P* = 0.05, partial *η* ^2^ = 0.03
RTs at 2‐back level	632.03 (131.13)	587.23 (116.94)	*F*_**1, 124**_ = 1.21., *P* = 0.27, partial *η* ^2^ = 0.01

Abbreviations: N/A, not applicable; RTs, reaction times.

^a^ These are the uncorrected values of neuropsychological and neurobehavioral testing and N‐back working‐memory performance.

* *P* ≤ 0.05.

** *P* ≤ 0.005.

*** *P* ≤ 0.001.

### Psychiatric measures

Within KUs, higher average ketamine dose was positively correlated with greater overall psychological distress (GSI; r = 0.343, *P* = 0.011) and with the anxiety (r = 0.457, *P* < 0.001, Bonferroni‐corrected *P* < 0.05) and hostility (r = 0.442, *P* < 0.001, Bonferroni‐corrected *P* < 0.05) subscales. Higher total ketamine use over the past month was also associated with higher GSI (r = 0.295, *P* = 0.030) and hostility scores (r = 0.396, *P* = 0.003, Bonferroni‐corrected *P* < 0.05). Correlations with other SCL‐90‐R subscales did not remain significant after Bonferroni correction. No significant correlations were observed for use frequency in the past 30 days or estimated duration since first exposure. Detailed SCL‐90 subscale for KUs are presented in Results Section S2, Tables S1 and Figure S1.

### Neuropsychological measures

No significant between‐group differences were observed across self‐reported psychological or behavioral measures (Table 1). Specifically, KUs and TUs did not differ on measures of overall psychological distress, impulsivity, aggression or sensitivity to punishment and reward. Detailed comparisons of SCL‐90 subscales are provided in Results Section S3 and Tables S2–S3.

### Working memory performance

For RTs, no significant main effect of group was observed (*F*_3, 122_ = 1.32, *P* = 0.27, partial η^2^ = 0.03), and the two groups showed comparable response speed across all load levels (0‐back, 1‐back, and 2‐back; all *P* > 0.05).

For accuracy, a significant main effect of group was detected (*F*_3, 122_ = 2.91, *P* = 0.037, partial η^2^ = 0.07). *Post hoc* comparisons indicated that KUs demonstrated lower accuracy than TUs under higher load conditions, including the 2‐back and 1‐back levels. Both groups showed the expected decrease in accuracy with increasing task load (0‐back > 1‐back > 2‐back; all *P* < 0.001) (Table 1).

These findings indicate that while processing speed was comparable between groups, KUs exhibited reduced accuracy under higher working‐memory demands. Detailed statistic information is presented in Results Section S4.

### Hippocampal volumes

A significant group difference was observed in left hippocampal volume. KUs showed smaller left hippocampal volumes compared with TUs. At the subfield level, KUs exhibited reduced volume in the left HATA relative to TUs. No other hippocampal subfields survived Bonferroni correction. Detailed subfield statistics are presented in Table 2.

**TABLE 2:** Hippocampal volumes by group.

Region	Group	Mean	SD	Mean (corrected)	SE	95% CI		*F* ^a^	Sig.	Partial *η* ^2^	Observed power
Left hippocampus	**KU**	**3451.65**	**308.52**	**3458.49**	**36.76**	**3385.70**	**3531.28**	**4.23**	**0.042** *	**0.03**	**0.53**
	TU	3570.13	324.63	3565.00	31.11	3503.40	3626.60				
Right hippocampus	KU	3535.67	315.80	3535.17	38.59	3458.75	3611.59	1.72	0.192	0.01	0.26
	TU	3606.20	331.91	3606.58	32.66	3541.91	3671.25				
Hippocampal subfield volumes^b^											
Left parasubiculum	KU	60.28	10.37	60.82	1.42	58.01	63.64	4.23	0.042	0.03	0.53
	TU	65.36	9.67	64.95	1.20	62.56	67.33				
Left presubiculum	KU	304.37	36.12	307.81	4.94	298.03	317.59	3.77	0.054	0.03	0.49
	TU	323.92	39.48	321.34	4.18	313.06	329.62				
Left subiculum	KU	438.19	48.86	442.67	6.46	429.88	455.47	1.79	0.183	0.02	0.26
	TU	458.23	53.60	454.87	5.47	444.04	465.70				
Left CA1	KU	616.63	66.64	616.15	8.58	599.16	633.15	2.09	0.151	0.02	0.30
	TU	633.30	74.53	633.65	7.26	619.27	648.03				
Left CA3	KU	201.05	24.72	198.64	3.21	192.28	205.01	0.90	0.346	0.01	0.16
	TU	201.12	24.71	202.93	2.72	197.55	208.32				
Left CA4	KU	243.83	22.74	243.34	2.78	237.82	248.85	2.07	0.153	0.02	0.30
	TU	248.62	23.69	248.99	2.36	244.32	253.65				
Left GC‐ML‐DG	KU	285.23	26.10	284.81	3.17	278.54	291.08	3.06	0.083	0.03	0.41
	TU	292.31	27.99	292.63	2.68	287.32	297.93				
Left molecular layer	KU	549.81	52.40	551.70	6.40	539.03	564.38	1.61	0.208	0.01	0.24
	TU	564.55	55.07	563.13	5.42	552.41	573.86				
Left HATA	**KU**	**56.54**	**8.85**	**55.66**	**1.11**	**53.47**	**57.85**	**10.52**	**0.002** **	**0.08**	**0.90**
	**TU**	**60.06**	**8.20**	**60.72**	**0.94**	**58.87**	**62.58**				
Left fimbria	KU	87.42	13.74	89.38	2.20	85.02	93.74	1.57	0.212	0.01	0.24
	TU	94.74	18.54	93.27	1.86	89.58	96.96				
Left tail	KU	608.31	65.45	607.49	10.26	587.17	627.81	2.12	0.148	0.02	0.30
	TU	627.91	75.58	628.53	8.68	611.33	645.72				
Left fissure	KU	144.30	25.72	144.17	3.52	137.19	151.14	0.25	0.618	0.00	0.08
	TU	141.58	24.20	141.68	2.98	135.78	147.59				
Right parasubiculum	KU	55.91	9.46	56.34	1.35	53.67	59.01	3.24	0.075	0.03	0.43
	TU	60.08	8.62	59.76	1.14	57.50	62.01				
Right presubiculum	KU	296.17	34.85	296.83	4.68	287.57	306.09	4.61	0.034	0.04	0.57
	TU	311.47	38.19	310.98	3.96	303.14	318.81				
Right subiculum	KU	438.84	46.39	439.49	6.27	427.08	451.89	3.89	0.051	0.03	0.50
	TU	457.38	53.79	456.90	5.30	446.40	467.39				
Right CA1	KU	657.78	72.22	657.96	10.05	638.06	677.86	0.60	0.440	0.01	0.12
	TU	669.08	79.33	668.95	8.51	652.10	685.79				
Right CA3	KU	212.20	29.37	211.05	3.92	203.29	218.80	0.26	0.608	0.00	0.08
	TU	207.34	27.90	208.21	3.32	201.64	214.77				
Right CA4	KU	246.08	24.19	245.41	3.01	239.45	251.38	0.18	0.671	0.00	0.07
	TU	246.72	23.71	247.22	2.55	242.17	252.27				
Right GC‐ML‐DG	KU	290.08	26.91	289.59	3.48	282.70	296.48	0.31	0.579	0.00	0.09
	TU	291.95	28.80	292.32	2.95	286.48	298.15				
Right molecular layer	KU	568.85	54.13	569.13	7.04	555.19	583.07	1.30	0.257	0.01	0.21
	TU	580.65	58.14	580.44	5.96	568.64	592.24				
Right HATA	KU	55.72	7.85	55.44	1.12	53.22	57.65	1.64	0.203	0.01	0.25
	TU	57.24	8.16	57.46	0.95	55.58	59.33				
Right fimbria	KU	77.85	14.45	78.64	2.28	74.12	83.17	4.04	0.047	0.03	0.51
	TU	85.71	17.70	85.11	1.93	81.29	88.94				
Right tail	KU	636.21	84.02	635.30	11.07	613.38	657.22	0.06	0.800	0.00	0.06
	TU	638.57	75.27	639.25	9.37	620.70	657.80				
Right fissure	KU	139.32	25.66	140.00	3.42	133.24	146.76	1.28	0.260	0.01	0.20
	TU	135.05	23.65	134.55	2.89	128.82	140.27				

Abbreviations: ANCOVA, Analysis of covariance; FTND, Fagerström Test for Nicotine Dependence; GC‐ML‐DG, granule cell and molecular layer of the dentate gyrus; HATA, hippocampal‐amygdaloid‐transition‐area; KU, smoking‐administered recreational ketamine use; Sig.: significant *P*; TIV, total intracranial volume; TU, tobacco use and without ketamine use.

^a^ANCOVA across ketamine use group, smoke use group after adjustment for age, sex, education year, FTND and TIV.

^b^Bonferroni correction is used to address multiple comparisons, setting the α level at 0.05/24 = 0.002.

Bold values indicate statistically significant results (*p* ≤ 0.05).

* *P* ≤ 0.05 (uncorrected, applies to left and right hippocampus).

** Bonferroni‐corrected *P* ≤ 0.05 (*P* = 0.05 / 24 = 0.002, applies to hippocampal subfields).

### Hippocampal functional connectivity

**FIGURE 1:**
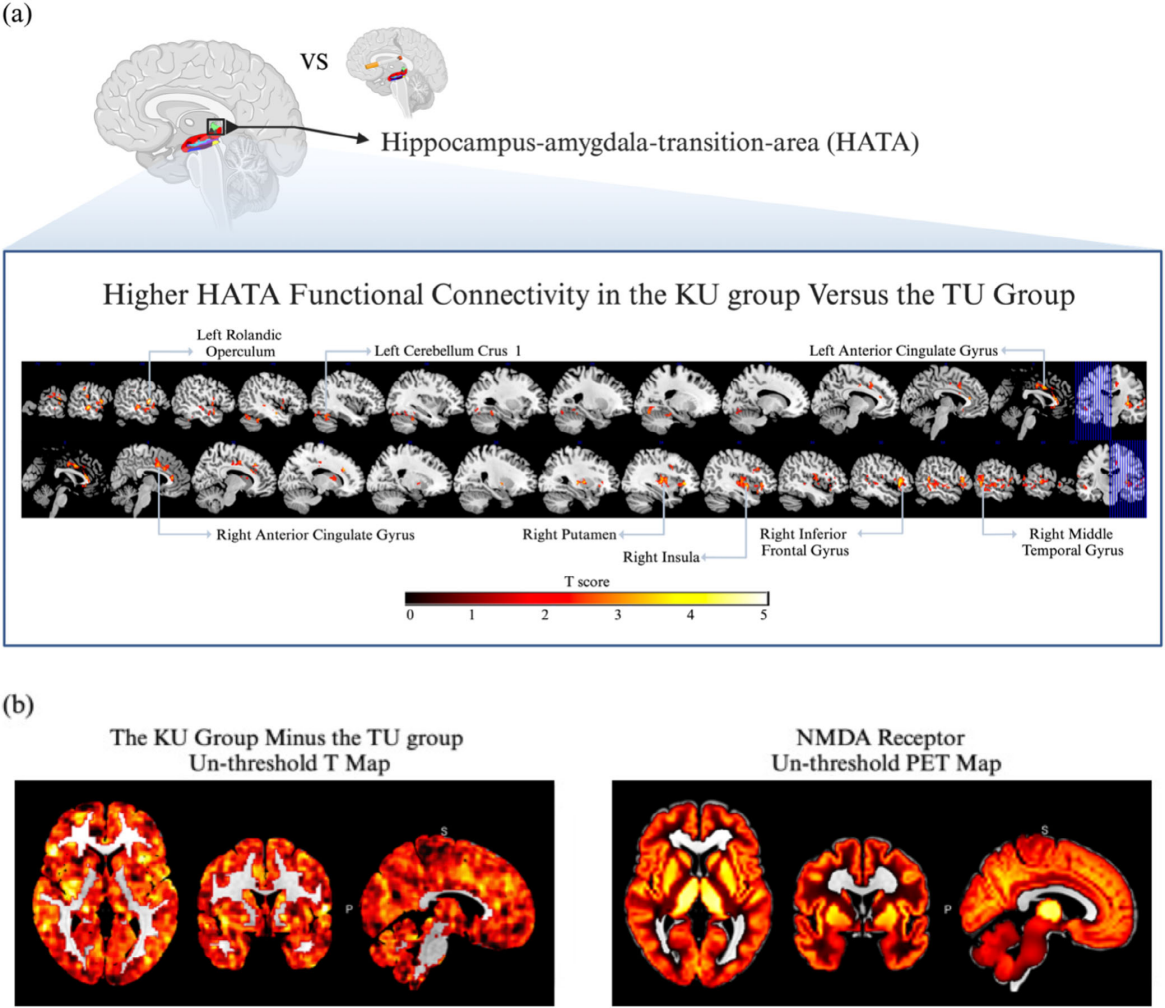
(a) The brain regions showing significant functional connectivity (FC) differences between smoking‐administered recreational ketamine use (KUs) and tobacco use and without ketamine use (TUs). The results indicate that KUs have greater left hippocampal‐amygdaloid‐transition‐area (HATA) connectivity with the left Rolandic operculum, right inferior frontal gyrus (triangular part), right inferior frontal gyrus (orbital part), left/right anterior cingulate and paracingulate gyri, right middle temporal gyrus, right lenticular nucleus (putamen), right insula, left cerebellum crus 1 and left fusiform gyrus (*P* < 0.05, cluster size > 462, AlphaSim corrected p_alpha_ < 0.05). The analyses were corrected for age, sex, years of education, Fagerström Test for Nicotine Dependence (FTND) and total intracranial volume (TIV) as covariates. (b) The maps show FC differences (KUs vs. TUs, unthresholded T map, left) and N‐methyl‐D‐aspartate (NMDA) receptor distribution [unthresholded positron emission tomography (PET) map, right]. Figures were created with BioRender.com.

Based on the observed group difference in hippocampal structure, the left HATA was selected as the seed region for FC analyses. Compared with TUs, KUs exhibited significantly greater FC between the left HATA and several cortical and subcortical regions, including the left Rolandic operculum, right inferior frontal gyrus (triangular and orbital parts), bilateral anterior cingulate and paracingulate gyri, right middle temporal gyrus, right lenticular nucleus (putamen), right insula, left cerebellar crus I and left fusiform gyrus (cluster‐level AlphaSim corrected, *P* < 0.05; cluster size ≥462 voxels). Corresponding statistical details and cluster coordinates are presented in Table 3, and spatial maps are shown in Figure 1(a).

**TABLE 3:** Higher left HATA functional connectivity in KUs compared to TUs.

MNI atlas coordinates			Voxel size	AAL	Nearest BA	T value
X	Y	Z				
−56	0	8	627	Left Rolandic operculum	BA 48	4.78
56	24	10	588	Right inferior frontal gyrus, triangular part/inferior frontal gyrus, orbital part	BA 45	4.34
−4	34	14	738	Left/right anterior cingulate and paracingulate gyri	BA 24	3.89
56	−44	10	746	Right middle temporal gyrus	BA 21	3.79
32	−20	2	620	Right lenticular nucleus, putamen/right insula	BA 48	3.76
−44	−70	−20	494	Left cerebellum crus 1/left fusiform gyrus	BA 19	3.31

Abbreviations: AAL, Automated Anatomical Labeling; BA, Brodmann area; HATA, hippocampal‐amygdaloid‐transition‐area; KUs, smoking‐administered recreational ketamine use; MNI, Montreal Neurological Institute; TUs, tobacco use and without ketamine use.

### Neurochemical correlates of hippocampal connectivity

**FIGURE 2:**
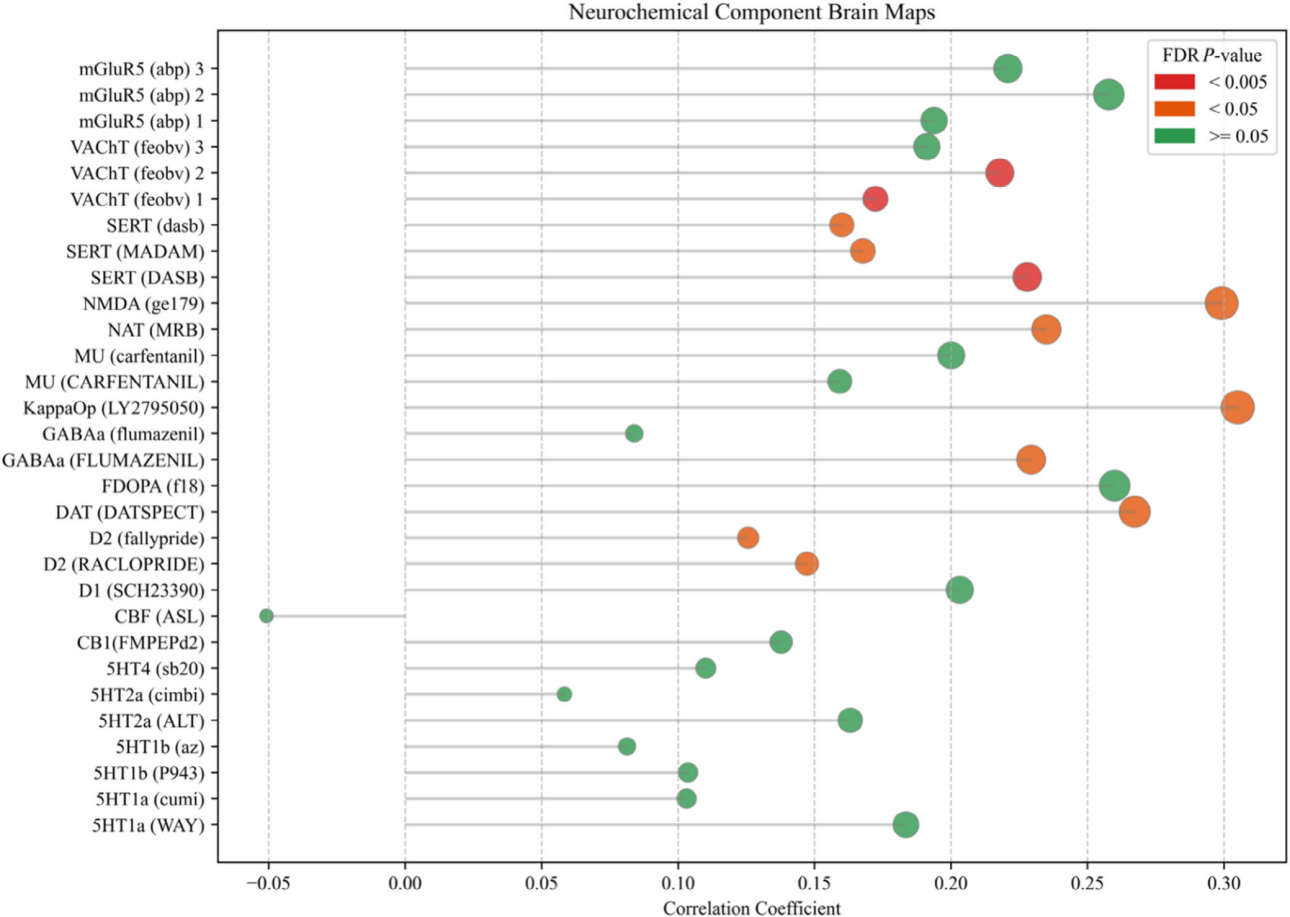
A lollipop chart showing the correlation coefficient (Fisher's z values) and the significant neurochemical component positron emission tomography (PET) maps. The *P*‐values have been false discovery rate (FDR) corrected to Benjamini‐Hochberg‐adjusted *P*‐values.

Spatial correlation analysis using JuSpace indicated that the KU > TU left HATA FC contrast map was positively associated with several neurotransmitter receptor and transporter distribution maps. Significant positive correlations (FDR‐corrected *P* < 0.05) were observed with serotonergic measures [SERT; 3‐amino‐4‐(2‐dimethylaminomethylphenylsulfanyl)‐benzonitrile (DASB): z = 0.23, *P* < 0.001; N, N‐dimethyl‐2‐(2‐amino‐4‐methylphenylthio) benzylamine (MADAM): z = 0.17, *P* = 0.001], VAChT [fluoroethoxybenzovesamicol (FEOBV): z = 0.22, *P* < 0.001; FEOBV replication map: z = 0.17, *P* < 0.001], NAT [methylreboxetine (MRB): z = 0.23, *P* = 0.001], dopaminergic measures (D2 receptor; raclopride: z = 0.15, *P* = 0.002; D2 receptor; fallypride: z = 0.13, *P* = 0.009; DAT; DATSPECT: z = 0.27, *P* = 0.007), glutamatergic NMDA receptor binding (GE179: z = 0.30, *P* = 0.005), κ‐opioid receptor (LY2795050: z = 0.30, *P* = 0.014) and GABA_A receptor distribution (flumazenil: z = 0.23, *P* = 0.015). These results indicate that regions showing greater left HATA connectivity in KUs exhibit spatial correspondence with multiple neuromodulatory systems [Figure 1(b), Figure 2; Results Section S5, Figure S2].

### *Post hoc* robustness analyses

To evaluate the robustness of the observed hippocampal structural findings, *post hoc* sensitivity analyses were conducted (see Section S3). The group differences in left hippocampal volume and the left HATA subfield remained significant following propensity‐score matching and when including psychiatric symptom severity (GSI) as a covariate. Group × sex interactions were not significant, while a group × FTND interaction indicated that nicotine dependence moderated hippocampal volume differences across groups. Within KUs, neither ketamine use frequency nor duration was associated with left hippocampal or HATA volume. The group effects also persisted when additionally controlling for daily cigarette use, age of smoking onset and ASI alcohol/drug composite scores. Overall, these analyses confirmed that the smaller volumes in the left hippocampus and HATA in KUs versus TUs were robust to demographic, psychiatric and substance–use‐related potential confounding factors. Detailed statistic information is presented in Results Section S6 and Tables S4–S13.

## DISCUSSION

### Overview

This study investigated differences in hippocampal structure and FC in KUs and TUs, addressing gaps in prior research that has largely relied on animal models or been limited by considerable polydrug use among humans. By examining individuals with well‐characterized ketamine‐use histories, we provide novel insight into subfield‐specific hippocampal structural differences associated with ketamine use. First, we characterized patterns of ketamine use and found that greater use was associated with increased psychiatric symptom severity, particularly anxiety and hostility. Second, although overall self‐reported affective and behavioral tendencies did not differ between groups, KUs exhibited reduced accuracy under high working‐memory load, suggesting specific cognitive impairment. Third, structural MRI analyses revealed a smaller volume in the left hippocampus, with the most pronounced difference observed in the HATA. Finally, FC analyses using the left HATA as a seed showed relatively increased connectivity with frontal, cingulate, temporal and subcortical regions in KUs, and these patterns spatially corresponded with neurotransmitter receptor distribution profiles, including NMDA receptor density. Together, these findings suggest that recreational ketamine use is associated with smaller hippocampal volumes, most substantially in the left HATA, in a manner associated with FC differences linked to NMDA and other neurotransmitter distributions.

### Psychiatric and cognitive characteristics

#### Substance use and psychiatric characteristics

Participants with longer estimated lifetime durations since first exposure to ketamine tended to use higher doses at one time and have a greater total amount of ketamine used in the last month. Based on serial investigations into non‐medicinal ketamine use, our results resonate with prior findings suggesting that tolerance may link to escalating use [10, 11, 55, 56, 57]. A positive correlation was observed between the recent usage dose of ketamine and scores on the SCL‐90, a widely used assessment tool for psychiatric symptomatology. Subjects with higher doses of recent use reported more overall psychiatric symptoms, resonating with previous studies in people with problematic use of heroin [58], ecstasy [59] and alcohol [60]. Anxiety and hostility concerns appeared most robustly linked to ketamine use. KUs who used higher doses, rather than those with longer use duration or higher frequency, exhibited more psychiatric symptomatology. This possible dose–response relationship aligns with studies indicating that ketamine's acute pharmacological effects are dose‐dependent and may be associated with transient psychiatric symptoms [13]. Regarding anxiety, the findings resonate with prior studies of heroin misuse that found positive correlations between negative emotions and drug use levels [58]. Considering the potential clinical symptoms, individuals with ketamine misuse may benefit from strengthening their abilities/tendencies to regulate negative emotions, which may also be linked to problems with hostility, although other possibilities (e.g. behavioral dysregulation when using ketamine) also warrant consideration.

#### Working memory

The findings suggest a load‐dependent working‐memory deficit in KUs, for example, accuracy declined selectively under higher working‐memory load (2‐back), while lower‐load performance remained intact. This pattern is consistent with clinical and neuroimaging evidence suggesting that ketamine‐related cognitive dysfunction is not global, but may instead disproportionately affect working memory and related processes. Acute ketamine impairs working‐memory manipulation and episodic encoding in healthy individuals [61], and long‐term heavy use has been linked to spatial‐memory concerns accompanied by reduced hippocampal and parahippocampal activation during memory‐guided navigation [62]. Our earlier study also observed poorer working‐memory performance in KUs relative to TUs [23], and the present findings specify that this impairment may become evident under higher working‐memory load. Executive dysfunction among KUs also has clinical implications, predicting reduced treatment engagement and increased dropout rates during detoxification [54]. Importantly, cognitive impairments may not be permanent, and longitudinal evidence indicates improvements in verbal and visual memory, as well as executive control, after approximately 12 weeks of abstinence [63].

Mechanistic evidence from animal research suggests that ketamine disrupts working memory by altering communication within prefrontal–hippocampal networks and shifting the balance between excitatory and inhibitory neural activity. In rhesus macaques, subanesthetic ketamine selectively impairs working memory while sparing sensory and motor functions, and this effect corresponds to reduced fast‐spiking interneuron activity, increased pyramidal‐cell firing in the lateral prefrontal cortex and a reduction in neural tuning precision [64]. In mice, a single ketamine exposure increases glutamatergic input from the prefrontal cortex to the nucleus reuniens and elevates firing of reuniens neurons, producing spatial working‐memory deficits, whereas (2R,6R)‐hydroxynorketamine modifies neuronal excitability without impairing working memory [65]. More broadly, NMDA receptor antagonists disrupt low‐γ synchronization across prefrontal–hippocampal circuits during memory maintenance, suggesting that working memory depends on precisely timed network coordination [66]. The temporal course of ketamine's effects is also relevant, for example, in a Wistar Kyoto model, clinically relevant ketamine doses initially impair working memory, but later enhance performance, reflecting potential neuroadaptive or compensatory processes that emerge outside the acute phase [67].

Overall, our observation of a possible selective deficit at higher working‐memory load is consistent with a model in which recreational ketamine use disrupts NMDA–receptor‐dependent communication between the prefrontal cortex and hippocampus and alters the balance between cortical excitation and inhibition, increasing working‐memory demands under more challenging cognitive conditions. Clinically, these findings support early cognitive screening and targeted interventions for KUs, including treatment approaches that address co‐occurring tobacco use where applicable. At the same time, the potential for cognitive recovery with sustained abstinence underscores the value of timely identification and support [61, 63, 67].

### Recreational ketamine use and hippocampal volume

KUs demonstrated smaller hippocampal volumes, consistent with pre‐clinical studies suggesting potential risks to hippocampal structure. Pre‐clinical studies have reported dose‐dependent associations between ketamine exposure and hippocampal tissue changes. In rats, higher ketamine doses were associated with reduced pyramidal and granule neurons and decreased glial cells within the molecular layer [68]. Similar hippocampal alterations were observed in adolescent cynomolgus monkeys following chronic subanesthetic ketamine exposure [69]. These pre‐clinical findings suggest potential relationships between ketamine exposure and hippocampal structural changes, possibly providing mechanistic context for the current findings.

Our findings in KUs may differ from clinical populations. In patients with major depressive disorder (MDD) receiving prescribed ketamine treatment, studies have reported volume increases in subcortical regions, potentially reflecting therapeutic neuroplasticity [70, 71]. However, our study of KUs observed smaller hippocampal volumes, particularly on the left side. Notably, these populations differ in multiple ways, for instance, dosing patterns, co‐occurring substance use, underlying psychiatric conditions and treatment context. Clinical studies involve controlled dosing in supervised medical settings for patients with depression, while our sample consisted of community‐recruited participants with recreational use and variable and often higher exposures to ketamine. Therefore, findings from KUs should not be directly extrapolated to clinical ketamine treatment for depression and vice versa.

Our study found that KUs had smaller left hippocampal volumes, particularly in the HATA. The HATA has been implicated in multiple neuropsychiatric conditions including MDD and post‐traumatic stress disorder [72]. The HATA is situated between the hippocampus and the amygdala. The hippocampus is primarily responsible for the formation and retrieval of memories, while the amygdala is critical in processing emotions, particularly those related to fear and pleasure [73]. Smaller HATA volumes in KUs may involve ketamine‐induced neurotoxicity, leading to neuronal death, reduced synaptic density and overall atrophy in this region. However, this possibility is currently speculative given the cross‐sectional nature of this study.

As smaller HATA volumes could suggest broader disruptions in neural networks involving the hippocampus and amygdala, we explored FC using the left HATA region as a seed. We observed relatively increased left‐HATA‐related connectivity to multiple brain regions in KUs. The identified brain regions link to multiple networks associated with psychiatric disorders like addictions and include the reward network [involving the inferior frontal gyrus and anterior cingulate cortex (ACC)], the salience network (involving the insula and ACC), the habit network (involving the putamen) and memory (involving the hippocampus) [74].

Additionally, PET maps suggest the possible involvement of glutamatergic and other neurotransmitter systems. Based on ketamine's glutamatergic effects, an intriguing finding is the relationship between FC differences and distribution of the NMDA receptor [75, 76]. Additionally, FC differences correlated with distributions of the κ‐opioid receptor, dopamine D2‐like receptors and dopamine transporter. The κ‐opioid receptor has been implicated in the regulation of reward and stress, dopaminergic systems [77] and substance addictions [77, 78, 79]. Additional results related to SERTs were unexpected. A previous human PET study found no measurable occupancy of the SERT after an antidepressant dose of ketamine, suggesting that ketamine's primary antidepressant mechanism might not involve SERT binding at standard doses despite a positive relationship between plasma levels and SERT occupancy [80]. As such, more study is needed to investigate neurochemical systems directly related to ketamine use.

### Robustness of structural findings

*Post hoc* sensitivity analyses provided support for the robustness of group differences in hippocampal structure. Even after propensity‐score matching on key demographic variables and controlling for nicotine dependence, psychiatric symptoms and broader substance use severity, KUs consistently exhibited smaller volumes in the left hippocampus and, more specifically, the HATA. These effects remained significant across multiple analytic conditions and were not moderated by sex or general psychiatric burden. Moreover, volumetric differences were not explained by recent ketamine use frequency or cumulative exposure duration, or by tobacco use intensity. We acknowledge that polydrug use can influence brain structure and function. However, the robustness of our findings after adjusting for alcohol‐ and drug‐related problem severity supports the specificity of ketamine‐related relationships with hippocampal measures. Together, these findings suggest that the observed hippocampal differences in KUs are not attributable to common demographic or clinical confounders and may reflect relatively stable, long‐term neurobiological consequences of ketamine use, although longitudinal studies are needed.

## CONCLUSIONS AND STUDY IMPLICATIONS

This study recruited young KUs from the general community who had no prior treatment history, with TUs serving as a comparison group to reduce confounding from nicotine‐related effects. We found that chronic recreational ketamine use was associated with region‐specific hippocampal differences, particularly within the HATA subfield, alongside altered FC in limbic–prefrontal circuits and networks linked to NMDA‐receptor and other systems. These findings linked to elevated psychiatric symptoms and worse load‐dependent working‐memory performance, aligning with clinical features observed in individuals with ketamine use disorder.

Given that many individuals with ketamine use disorder do not seek treatment because of limited effective interventions [81], our finding that negative affect relates to levels of ketamine use highlights emotion regulation as a potential therapeutic target. Cognitive‐behavioral therapy oriented toward emotion regulation has demonstrated benefit in treating substance use disorders among young adults [82] and may be relevant for ketamine‐related difficulties. Furthermore, hippocampal structural and functional differences may serve as clinically useful biomarkers for risk evaluation and treatment monitoring. The involvement of NMDA‐related pathways also suggests a rationale for precise interventions, such as guided neuromodulation [83]. Together, these results underscore the need for neurobiological informed, multi‐modal interventions targeting both cognitive and affective dysregulation in ketamine use.

### Limitations

This study has several limitations. First, the cross‐sectional design precludes causal inference, and longitudinal studies are needed to establish temporal relationships. Second, ketamine use was self‐reported without biochemical verification and variability in the composition of recreational ketamine limits standardized dosing estimates. Third, demographic differences between groups were addressed statistically, but residual confounding cannot be fully excluded. Fourth, we did not directly assess craving or withdrawal, which represent meaningful clinical features that warrant examination in future work. Finally, although TUs served as a comparison group to reduce nicotine‐related confounding, ketamine and tobacco use frequently co‐occur in real‐world contexts. Future work should also include individuals with ketamine use and without tobacco exposure to more clearly isolate ketamine‐specific neurocognitive effects and refine biomarker development and intervention strategies.

## AUTHOR CONTRIBUTIONS

**Yi‐Hsuan Liu:** Conceptualization (equal); data curation (lead); formal analysis (lead); funding acquisition (supporting); methodology (equal); visualization (lead); writing—original draft (lead). **Chia‐Chun Hung:** Conceptualization (equal); data curation (equal); funding acquisition (equal); investigation (equal); methodology (equal); resources (equal); writing—review and editing (equal). **Marc N. Potenza:** Investigation (equal); writing—original draft (equal); writing—review and editing (lead). **Kun‐Hsien Chou:** Conceptualization (equal); funding acquisition (supporting); methodology (equal); writing—review and editing (supporting). **Pei‐Lin Lee:** Methodology (supporting); software (supporting); writing—review and editing (supporting). **Chu‐Chung Huang:** Conceptualization (supporting); data curation (supporting). **Chiang‐Shan R. Li:** Formal analysis (equal); investigation (supporting); resources (supporting); software (supporting). **Tony Szu‐Hsien Lee:** Conceptualization (equal); funding acquisition (lead); investigation (lead); resources (lead); supervision (lead); writing—review and editing (lead). **Ching‐Po Lin:** Conceptualization (equal); funding acquisition (lead); investigation (equal); project administration (supporting); resources (lead); supervision (lead); writing—review and editing (lead).

## DECLARATION OF INTERESTS

The authors have no conflicts of interest to report. M.N.P. discloses the following: he has consulted for Neurofinity and Boehringer Ingelheim; has been involved in a patent application with Yale University and Novartis; has received research support from Mohegan Sun Casino and the Connecticut Council on Problem Gambling; has participated in surveys, mailings or telephone consultations related to drug addiction, internet use, impulse‐control disorders or other health topics; has consulted for and/or advised gambling, non‐profit, healthcare and legal entities on issues related to internet use, impulse control and addictive disorders; has performed grant reviews for research‐funding agencies; has edited journals and journal sections; has given academic lectures in grand rounds, CME events and other clinical or scientific venues; and has generated books or book chapters for publishers of mental health texts. The other authors have no disclosures to report.

## Supporting information


**Table S1.** The results of the Symptom Checklist‐90 for participants who use ketamine.
**Table S2**. ANCOVA Summary for the Symptom Checklist‐90 Total Score and General Severity Index between KU participants and TU participants.
**Figure S1. The results of the Symptom Checklist‐90 for participants who use ketamine.** (a) The box and whisker plot showing KU participants' SCL‐90 quartile distribution for the total score and General Severity Index (GSI). (b) Showing dimension scores including Somatization (SOM), Obsessive‐compulsive (O‐C), Interpersonal sensitivity (I‐S), Depression (DEP), Anxiety (ANX), Hostility (HOS), Phobic anxiety (PHOB), Paranoid ideation (PAR), and Psychoticism (PSY).
**Table S3.** ANCOVA Summary for the Symptom Checklist‐90 Sub‐items between KU participants and TU participants.
**Figure S2.** Left HATA FC T‐Map and PET Maps Results. (a) The correlations of mean values between Left HATA FC data and each neurochemical component PET map, applying 442 regional brain component PET maps. (b) The bar chart showing the correlation coefficient (Fisher's z values) and the significant PET maps. The p‐values are the original p‐values. * Indicates FDR corrected p‐value < 0.05.
**Table S4**. ANCOVA Summary for Whole Hippocampal Volume between KU participants and TU participants (Matched Sample, n = 51 per group).
**Table S5.** ANCOVA Summary for Hippocampal Subfields Volume between KU participants and TU participants (Matched Sample, n = 51 per group).
**Table S6.** ANOVA Summary for Whole Hippocampal Volume between KU participants and TU participants (Matched Sample, n = 31 per group).
**Table S7.** ANCOVA Summary for Hippocampal Subfields Volume between KU participants and TU participants (Matched Sample, n = 31 per group).
**Table S8.** ANOVA Summary for Whole Hippocampal Volume between KU participants and TU participants (Psychiatric Symptoms).
**Table S9.** ANCOVA Summary for Hippocampal Subfields Volume between KU participants and TU participants (Psychiatric Symptoms).
**Table S10.** ANOVA Summary for Whole Hippocampal Volume between KU participants and TU participants (Nicotine‐Related Variables).
**Table S11.** ANCOVA Summary for Hippocampal Subfields Volume between KU participants and TU participants (Nicotine‐Related Variables).
**Table S12.** ANOVA Summary for Whole Hippocampal Volume between KU participants and TU participants (Broader Substance Use).
**Table S13.** ANCOVA Summary for Hippocampal Subfields Volume between KU participants and TU participants (Broader Substance Use).

## Data Availability

The data that support the findings of this study are available on request from the corresponding author. The data are not publicly available due to privacy or ethical restrictions.
