# Supplementary material for: Hippocampal subfield differences in people with and without recreational ketamine use: Insights from multi‐modal neuroimaging
Source: Addiction. 2026 Jan 29;121(6):1541–56. doi: 10.1111/add.70331 (PMC13155325; doi:10.1111/add.70331)
Supplement: Supplementary file 1 — Table S1. The results of the Symptom Checklist‐90 for participants who use ketamine. Table S2. ANCOVA Summary for the Symptom Checklist‐90 Total Score and General Severity Index between KU participants and TU participants. Figure S1. The results of the Symptom Checklist‐90 for participants who use ketamine. (a) The box and whisker plot showing KU participants' SCL‐90 quartile distribution for the total score and General Severity Index (GSI). (b) Showing dimension scores including Somatization (SOM), Obsessive‐compulsive (O‐C), Interpersonal sensitivity (I‐S), Depression (DEP), Anxiety (ANX), Hostility (HOS), Phobic anxiety (PHOB), Paranoid ideation (PAR), and Psychoticism (PSY). Table S3. ANCOVA Summary for the Symptom Checklist‐90 Sub‐items between KU participants and TU participants. Figure S2. Left HATA FC T‐Map and PET Maps Results. (a) The correlations of mean values between Left HATA FC data and each neurochemical component PET map, applying 442 regional brain component PET maps. (b) The bar chart showing the correlation coefficient (Fisher's z values) and the significant PET maps. The p‐values are the original p‐values. * Indicates FDR corrected p‐value < 0.05. Table S4. ANCOVA Summary for Whole Hippocampal Volume between KU participants and TU participants (Matched Sample, n = 51 per group). Table S5. ANCOVA Summary for Hippocampal Subfields Volume between KU participants and TU participants (Matched Sample, n = 51 per group). Table S6. ANOVA Summary for Whole Hippocampal Volume between KU participants and TU participants (Matched Sample, n = 31 per group). Table S7. ANCOVA Summary for Hippocampal Subfields Volume between KU participants and TU participants (Matched Sample, n = 31 per group). Table S8. ANOVA Summary for Whole Hippocampal Volume between KU participants and TU participants (Psychiatric Symptoms). Table S9. ANCOVA Summary for Hippocampal Subfields Volume between KU participants and TU participants (Psychiatric Symptoms). Table S10. ANOVA Summar [file ADD-121-1541-s001.pdf]

SUPPLEMENTARY MATERIAL

Supplementary Methods

Section S1. N-back Working Memory Task

A sequential-letter version of the N-back working memory task adapted from Koshino et al. (2005) was used to assess attention and working memory capacity. The task consisted of three load levels (0-back, 1-back, and 2-back). Stimuli were drawn from a set of 16 consonant letters (A, B, C, D, E, F, G, H, K, M, N, P, S, W, X) presented one at a time in the center of the screen. Participants were instructed to press a response button whenever a target was detected. In the 0-back condition, the target was a pre-specified letter ("X"), requiring stimulus detection without working memory involvement. In the 1-back condition, the target was a letter identical to the one immediately preceding it. In the 2-back condition, the target was a letter identical to the one presented two positions earlier. Each block contained 23 letter stimuli, including 7 target trials pseudo randomly interspersed. Each stimulus appeared for 500 ms, followed by a 1500-ms fixation interval. Participants completed four blocks of each load level (0-back, 1-back, and 2-back), presented in a randomized order. Accuracy (%) and reaction time (RT, ms) for correct responses were recorded as performance measures.

Section S2. MRI data acquisition protocol

Imaging data were acquired at National Yang Ming Chiao Tung University using a 3.0-T Siemens Magnetom Tim Trio MRI scanner (Siemens, Erlangen, Germany) equipped with a 32-channel head coil. High-resolution T1-weighted structural images were obtained using a sagittal 3D magnetization-prepared rapid gradient-echo sequence (MPRAGE; TR = 3500 ms, TE = 3.5 ms, TI = 1100 ms, FOV = 256 mm, flip angle = 7°, matrix size = 256 × 256, 192 sagittal slices, voxel size = 1.0 × 1.0 × 1.0 mm). Resting-state fMRI data were acquired using a gradient echo single-shot echo planar imaging (EPI) sequence (TR = 2500 ms, TE = 27 ms, FOV = 220 mm, flip angle = 77°, matrix size = 64 × 64, 43 axial slices, voxel size = 3.4 × 3.4 × 3.4 mm, no gap) for a total duration of 8 minutes. During the scan, participants were instructed to keep their eyes closed, remain still, stay awake, and not engage in structured thoughts. Head motion was minimized using foam padding, and all scans were visually inspected by an experienced radiologist and trained research staff to ensure the absence of scanner artifacts, excessive motion, or gross anatomical abnormalities.

Supplementary Results

Section S1. Tobacco Use and Addiction Severity Index (ASI) Comparisons

Between-group comparisons of tobacco use characteristics indicated that ketamine use participants had significantly higher nicotine dependence scores on the Fagerström Test for Nicotine Dependence (KU: M = 5.18, SD = 2.39; TU: M = 3.78, SD = 2.42; mean difference = 1.41, 95% CI [0.57, 2.25], $t(129) = 3.32$, $p = 0.001$, Cohen's $d = 0.59$, 95% CI [0.23, 0.94]), greater daily cigarette consumption (KU : M = 19.62 , SD = 10.17, TU : M = 13.22 , SD = 8.33; mean difference = 6.40 cigarettes, 95% CI [3.12, 9.68], $t(129) = 3.870$, $p < 0.001$, Cohen's $d = 0.70$, 95% CI [0.34, 1.05]), and an earlier age of tobacco use onset (KU : M = 15.63 , SD = 2.40, TU : M = 17.87 , SD = 4.41; mean difference = -2.24 years, 95% CI [-3.52, -0.97], $t(129) = -3.478$, $p < 0.001$, Cohen's $d = -0.61$, 95% CI [-0.96, -0.26]). However, no significant group differences were found in tobacco use frequency (days/week; KU : M = 6.16 , SD = 2.07, TU : M = 5.69 , SD = 2.24; T = 1.24, $p = 0.219$; mean difference = 0.47, 95% CI [-0.28, 1.22], $t(129) = 1.236$, $p = 0.219$, Cohen's $d = 0.22$, 95% CI [-0.13, 0.56]) or in estimated lifetime duration since first tobacco use (KU : M = 5.37 , SD = 4.66 years, TU : M = 6.47 , SD = 5.51 years; mean difference = -1.10 years, 95% CI [-2.89, 0.69], $t(129) = -1.214$, $p = 0.227$, Cohen's $d = -0.21$, 95% CI [-0.56, 0.13]).

Addiction Severity Index composite scores differed significantly between groups for drug use severity. Ketamine use participants showed substantially higher drug composite scores than controls (KU: M = 0.06, SD = 0.08; TU: M = 0.006, SD = 0.02; mean difference = 0.06, 95% CI [0.04, 0.08], $t(129) = 6.001$, p < 0.001, Cohen's $d = 1.06$, 95% CI [0.69, 1.42]). No significant differences were observed for medical composite scores (KU: M = 0.09, SD = 0.20; TU: M = 0.11, SD = 0.21; mean difference = -0.02, 95% CI [-0.09, 0.05], $t(129) = -0.59$, p < 0.559, Cohen's $d = -0.10$, 95% CI [-0.49, 10.24]) or alcohol composite scores (KU: M = 0.12, SD = 0.13; TU: M = 0.12, SD = 0.11; mean difference = 0.0005, 95% CI [-0.04, 0.04], $t(129) = 0.02$, p < 0.981, Cohen's $d = 0.0004$, 95% CI [-0.34, 0.35])

Section S2. Symptom Checklist-90 for participants who use ketamine

Supplementary Table S1. The results of the Symptom Checklist-90 for participants who use ketamine.

	Min	Q1	Median	Q3	Max	Mean	SD
Total Score	90.00	93.25	122.00	172.00	270.00	141.24	56.01
General Severity Index, GSI	1.00	1.04	1.36	1.91	3.00	1.57	0.62
Dimension Scores							
Somatization	11.00	11.00	13.50	18.00	33.00	15.83	5.83
Obsessive-compulsive	10.00	11.25	15.50	21.00	35.00	16.93	6.46
Interpersonal sensitivity	9.00	9.00	11.50	19.00	33.00	14.48	6.42
Depression	13.00	13.00	17.50	27.50	44.00	21.40	9.93
Anxiety	10.00	10.00	11.50	20.00	34.00	15.45	6.77
Hostility	6.00	6.00	8.00	12.75	24.00	10.12	4.79
Photic anxiety	7.00	7.00	7.00	10.75	23.00	9.64	4.17
Paranoid ideation	6.00	6.00	7.50	11.00	21.00	9.38	4.32
Psychoticism	10.00	10.00	13.00	17.75	32.00	15.38	6.52

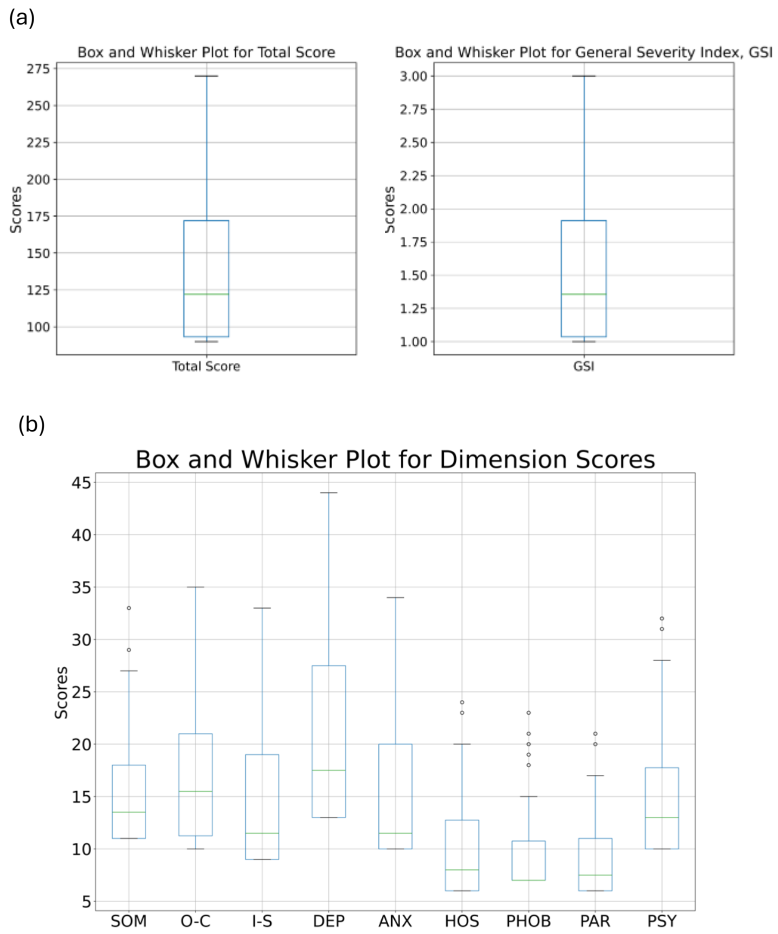

Figure S1: The results of the Symptom Checklist-90 for participants who use ketamine. (a) The box and whisker plot showing KU participants' SCL-90 quartile distribution for the total score and General Severity Index (GSI). (b) Showing dimension scores including Somatization (SOM), Obsessive-compulsive (O-C), Interpersonal sensitivity (I-S), Depression (DEP), Anxiety (ANX), Hostility (HOS), Phobic anxiety (PHOB), Paranoid ideation (PAR), and Psychoticism (PSY).

Section S3. Group Comparison on SCL-90-R Scores

An analysis of variance (ANOVA) was conducted to compare the SCL-90-R Total Score, General Severity Index (GSI), and nine subscale scores between KU participants and TU participants. No significant group differences were observed across any of the scales.

An analysis of variance (ANOVA) was conducted to compare Symptom Checklist-90 Total Score and General Severity Index, GSI separately between KU participants and TU participants. No significant group effect was found for the Symptom Checklist-90 Total Score and General Severity Index, GSI.

Supplementary Table S2. ANCOVA Summary for the Symptom Checklist-90 Total Score and General Severity Index between KU participants and TU participants

	Group	Mean	SD	Corrected Mean	SE	95% Confidence Interval		F	Sig.	Partial η^2	Observed Power
Total Score	KU	141.24	56.01	142.46	8.10	126.43	158.49	0.01	0.906	0.00	0.05
	TU	142.05	59.36	141.09	7.09	127.06	155.11				
General Severity Index, GSI	KU	1.57	0.62	1.58	0.09	1.41	1.76	0.02	0.887	0.00	0.05
	TU	1.58	0.66	1.57	0.08	1.41	1.72				

An analysis of variance (ANOVA) was conducted to compare Symptom Checklist-90 sub items between KU participants and TU participants. No significant group effect was found for the all sub items.

Supplementary Table S3. ANCOVA Summary for the Symptom Checklist-90 Sub-items between KU participants and TU participants

	Group	Mean	SD	Corrected Mean	SE	95% Confidence Interval		F	Sig.	Partial η²	Observed Power
Somatization	KU	15.83	5.83	15.99	0.89	14.23	17.74	0.09	0.772	0.00	0.06
	TU	16.48	6.91	16.35	0.78	14.82	17.89				
Obsessive-compulsive	KU	16.93	6.46	17.02	0.99	15.07	18.97	0.30	0.585	0.00	0.08
	TU	17.86	7.41	17.79	0.86	16.08	19.50				
Interpersonal sensitivity	KU	14.48	6.42	14.59	0.87	12.88	16.31	0.28	0.599	0.00	0.08
	TU	14.03	6.09	13.94	0.76	12.44	15.44				
Depression	KU	21.40	9.93	21.78	1.49	18.82	24.73	0.04	0.850	0.00	0.05
	TU	21.67	11.08	21.37	1.31	18.79	23.95				
Anxiety	KU	15.45	6.77	15.64	1.03	13.60	17.68	0.04	0.842	0.00	0.06
	TU	15.49	7.71	15.34	0.90	13.56	17.13				
Hostility	KU	10.12	4.79	9.99	0.72	8.57	11.41	0.05	0.830	0.00	0.06
	TU	10.11	5.29	10.21	0.63	8.97	11.46				
Photic anxiety	KU	9.64	4.17	9.63	0.56	8.52	10.74	0.37	0.543	0.00	0.09
	TU	9.14	3.96	9.14	0.49	8.17	10.11				
Paranoid ideation	KU	9.38	4.32	9.36	0.58	8.21	10.52	0.02	0.880	0.00	0.05
	TU	9.22	3.87	9.23	0.51	8.22	10.24				

Psychoticism	KU	15.38	6.52	15.68	0.93	13.84	17.52	0.56	0.454	0.00	0.12
	TU	14.92	6.63	14.68	0.82	13.07	16.29				

Section S4. N-back working memory task performance

For reaction times (RTs), the group-by-level interaction was insignificant ($F(\mathbf{1, 119})$ = 1.32, p = 0.269, partial η^2 = 0.03). The main effect showed that the two groups showed similar patterns regarding mean RTs. KU and TU participants did not show differences in RTs for 0-back (KU mean = 477.98 ± 86.55, TU mean = 449.64 ± 69.22, mean difference = 12.37, 95% CI [-19.13, 43.86], $F(\mathbf{1, 124})$ = 0.60., p = 0.44, partial η^2 = 0.005), 1-back (KU mean = 551.96 ± 102.75, TU mean = 497.92 ± 92.72, mean difference = 39.72, 95% CI [-0.32, 79.75], $F(\mathbf{1, 124})$ = 3.86., p = 0.05, partial η^2 = 0.03), or 2-back (KU mean = 632.03 ± 131.13, TU mean = 587.23 ± 116.94, mean difference = 27.52, 95% CI [-22.04, 77.08], $F(\mathbf{1, 124})$ = 1.21., p = 0.27, partial η^2 = 0.01) trials. For the TU group, RTs during 2-back trials were longer than 1-back trials (t = 8.66; p < 0.001) and 0-back trials (t = 12.61, p < 0.001). The RTs of 1-back trials were also longer during 0-back trials (t = 5.39, p < 0.001). Results were similar for KU participants: RTs during 2-back trials were longer than 1-back (t = 4.93, p < 0.001) and 0-back (t = 9.94, p < 0.001) trials and RTs during 1-back trials were longer than 0-back trials (t = 6.41, p < 0.001).

A group-by-level interaction effect for accuracy was observed ($F(\mathbf{1, 124})$ = 2.91, p = 0.037, partial η^2 = 0.07). The main effect of the group showed that KU participants showed lower accuracy than TU participants during 2-back (KU mean = 0.63 ± 0.24, TU mean = 0.76 ± 0.19, mean difference = -0.09, 95% CI [-0.18, -0.003], $F(\mathbf{1, 124})$ = 4.16., p = 0.04, partial η^2 = 0.03) and 1-back (KU mean = 0.77 ± 0.02, TU mean = 0.91 ± 0.13, mean difference = -0.09, 95% CI [-0.16, -0.02], $F(\mathbf{1, 124})$ = 8.10., p = 0.005, partial η^2 = 0.06) performance (See Table 1). Among TU participants, a main effect of level indicated that mean accuracy during 2-back performance was lower than during 1-back performance (t = -8.14, p < 0.001) and 0-back performance (t = -9.07, p < 0.001). The mean accuracy during 1-back performance was lower than during 0-back performance (t = -3.61, p = 0.001). For KU participants, the mean accuracy during 2-back performance was lower than during 1-back performance (t = -5.93, p < 0.001) and 0-back performance (t = -7.49, p < 0.001). The mean accuracy during 1-back performance was lower than during 0-back (t = -4.00, p < 0.001).

The KU group showed significantly lower accuracy in the high-load n-back condition, indicating that participants with KU demonstrated poorer working memory.

Section S5. Similarity comparison between Left HATA FC T-Map and PET maps results

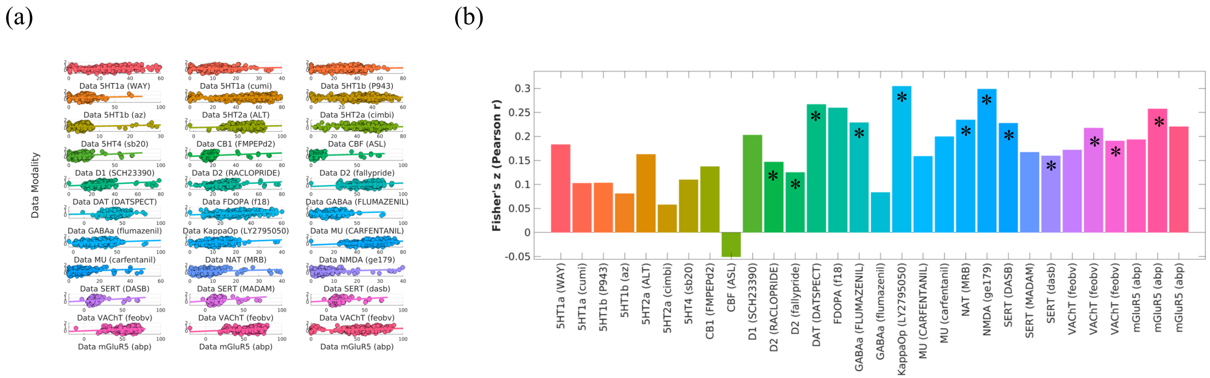

Figure S2: Left HATA FC T-Map and PET Maps Results. (a) The correlations of mean values between Left HATA FC data and each neurochemical component PET map, applying 442 regional brain component PET maps. (b) The bar chart showing the correlation coefficient (Fisher's z values) and the significant PET maps. The p-values are the original p-values. * Indicates FDR corrected p-value < 0.05.

Section S6. Post-Hoc Robustness Checks

To assess the robustness of our primary findings regarding hippocampal structural differences, we conducted a series of post hoc analyses. First, ANCOVAs were repeated in a propensity score–matched (PSM) subsample (n = 51 per group), controlling for age, sex, education years, and nicotine dependence (FTND scores). Results remained consistent with the full sample: significant group effects were observed for left hippocampal volume ($F(1, 92) = 6.89$, $p = .010$, partial $\eta^2 = .070$, observed power = .74) and the left hippocampal–amygdaloid transition area (HATA) subfield ($F(1, 92) = 13.10$, p < .001, partial $\eta^2 = .13$, observed power = .95), both of which survived Bonferroni correction. These results held when reanalyzed using a more stringent caliper of 0.2 (n = 31 per group), minimizing residual group differences in age and education. (Supplementary Tables S4–S7)

To further examine whether psychiatric symptoms influenced group effects, the Global Severity Index (GSI) from the SCL-90-R was added as a covariate. The group difference in left hippocampal volume remained significant ($F(1, 92) = 6.89$, $p = .010$), with a nonsignificant Group × GSI interaction ($F(1, 92) = 0.73$, $p = .395$), indicating that psychiatric burden did not moderate the group effect. A similar pattern was observed in the left HATA subfield (group: $F(1, 92) = 13.10$, p < .001; interaction: $F(1, 92) = 1.18$, $p = .281$). (Supplementary Tables S8–S9)

To explore moderation by sex and nicotine dependence, Group × Sex and Group × FTND interactions were tested. No significant sex interactions were found for either the left hippocampus ($F(1, 92) = 0.04$, $p = .838$) or left HATA ($F(1, 92) = 0.02$, $p = .887$). However, a significant Group × FTND interaction emerged for the left hippocampus ($F(1, 92) = 7.69$, $p = .007$, partial $\eta^2 = .077$), suggesting that nicotine dependence moderated hippocampal volume differently across groups. Follow-up correlations revealed a significant negative association in the TU group (r = –.330, $p = .018$) but a nonsignificant positive association in the KU group (r = .176, $p = .213$). No interaction was found for the HATA ($F(1, 92) = 1.35$, $p = .247$). (Supplementary Tables S8–S9)

We further explored whether hippocampal alterations were related to ketamine use intensity. Partial correlations within the KU group (n = 51), controlling for age, sex, education, FTND, and GSI, revealed no significant associations between left hippocampal volume and either use frequency (r = .07, $p = .626$) or duration (r = .09, $p = .542$). Similar nonsignificant results were found for the left HATA subfield (frequency: r = –.09, $p = .555$; duration: r = –.07, $p = .631$), suggesting that recent use patterns did not account for volumetric reductions.

Controlling for Nicotine-Related Variables

To more comprehensively address nicotine-related confounds, we included average daily cigarette consumption and age of tobacco onset as additional covariates in ANCOVA models. A significant group difference was observed in left hippocampal volume ($F(1, 117) = 6.73$, $p = .010$, partial $\eta^2 = .05$**,** observed power = .73), surviving Bonferroni correction (adjusted α = .025), but not for the right hippocampus ($F(1, 117) = 3.02$, $p = .085$). Left HATA differences remained significant ($F(1, 117) = 12.38$, $p < .001$, partial $\eta^2 = .09$, observed power = .94, adjusted α = .002).

To further explore whether tobacco use intensity explained group effects, partial correlations were conducted in KU and TU groups between hippocampal volume and tobacco use metrics (frequency and duration), controlling for age, sex, education, FTND, and TIV. No significant associations were observed in either group for the left hippocampus or HATA (all ps > .1). (Supplementary Tables S10–S11)

Controlling for Broader Substance Use

Lastly, ANCOVAs controlling for alcohol (ACOMP) and drug (DCOMP) composite scores from the Addiction Severity Index were performed. The group effect remained significant for the left hippocampus ($F(1, 117) = 5.83$, $p = .017$, partial $\eta^2 = .05$, observed power = .67) and left HATA ($F(1, 117) = 11.05$, $p = .001$, partial $\eta^2 = .09$, observed power = .91), both surviving Bonferroni correction. No group difference was found in right hippocampal volume ($F(1, 117) = 2.35$, $p = .128$). (Supplementary Tables S12–S13)

Collectively, these post hoc analyses confirm that group differences in hippocampal and subfield volumes, particularly in the left hemisphere, are robust to a range of demographic, psychiatric, and substance use confounders.

Post Hoc Sensitivity Analysis: Propensity Score Matching (Caliper = 0.4, n = 51 per group)

To reduce potential bias associated with baseline group differences, we conducted a post-hoc propensity score matching (PSM) analysis. A logistic regression model was used to estimate propensity scores based on age, sex, years of education, and nicotine dependence (FTND score). The outcome variable indicated group membership (1 = ketamine use [KU], 0 = tobacco use [TU]).

The overall model was significant (χ^2 (4) = 40.30, p < .001), with a Nagelkerke R^2 of .368, indicating a moderately strong prediction of group membership from the included covariates. Among the predictors, years of education emerged as the only statistically significant variable (B = –0.53, SE = 0.13, Wald = 16.03, p < .001), suggesting KU participants had significantly fewer years of education. Sex, age, and FTND were not significant predictors in this model.

Using a 1:1 nearest neighbor matching algorithm without replacement and a caliper width of 0.4, we successfully matched 51 KU participants to 51 TU participants. Three KU participants could not be matched and were excluded. After matching, the average number of available controls matches per case was 20.33, indicating adequate matching quality. However, small residual group differences in age and education persisted, and these covariates were subsequently controlled for in all post-matching analyses.

The matched sample showed improved balance in demographic characteristics, with increased classification accuracy in the logistic regression model (overall classification = 81.0%). The final matched sample was used for all subsequent ANCOVA analyses to evaluate group effects on hippocampal subfield volumes.

An analysis of covariance (ANCOVA) on bilateral whole hippocampal volumes

An analysis of covariance (ANCOVA) was conducted to compare bilateral whole hippocampal volumes between KU participants and TU participants in a matched sample (n = 51 per group), controlling for age and years of education.

A significant group effect was observed for the **left hippocampus (F (1, 98) = 7.08, *p* = .009, partial η^2 = .06, power = .75)**, which survived Bonferroni correction for multiple comparisons (adjusted α = .025). Post hoc pairwise comparisons (Bonferroni-adjusted) revealed a lower adjusted mean volume in KU group than in TU group. Specifically, the corrected mean (SE) volume of the left hippocampus was 3441.66 mm^3 (45.66) in the KU group and 3621.53 mm^3 (45.66) in the TU group. Confidence intervals indicated non-overlapping group estimates, supporting the robustness of the observed group difference.

Supplementary Table S4. ANCOVA Summary for Whole Hippocampal Volume between KU participants and TU participants (Matched Sample, n = 51 per group)

Region	Group	Mean	SD	Corrected	SE	95% CI	95% CI	F	p	partial	Observed

				Mean		Lower	Upper			η²	Power
**Left Hippocampus**	**KU**	**3456.11**	**316.34**	**3441.66**	**45.66**	**3351.05**	**3532.28**	**7.08**	**0.009****	**0.06**	**0.75**
	**TU**	**3607.09**	**332.44**	**3621.53**	**45.66**	**3530.91**	**3712.15**				
Right Hippocampus	KU	3543.69	321.71	3519.49	46.14	3427.92	3611.06	4.70	0.032	0.04	0.58
	TU	3643.46	341.48	3667.66	46.14	3576.09	3759.23				

Note. ANCOVA was conducted on a matched sample (n = 51 per group), controlling for age and years of education. Covariates were evaluated at the following values: age = 22.63 years, education = 10.73 years. Pairwise comparisons were Bonferroni-corrected for multiple comparisons across the left and right hippocampus (2 comparisons), with the alpha level set at .025 (0.05/2).
** Indicates Bonferroni-corrected $p < .05$ for bilateral hippocampal volume comparisons (adjusted alpha = .025).

An analysis of covariance (ANCOVA) on hippocampal subfield volumes

An analysis of covariance (ANCOVA) was conducted to compare bilateral whole hippocampal volumes between KU participants and TU participants in a matched sample (n = 51 per group), controlling for age and years of education.

A significant group effect was observed for **the left HATA ($F(1, 98) = 12.27$, $p < .001$, partial $\eta^2 = .11$, power = .93**, which survived Bonferroni correction for multiple comparisons (adjusted $\alpha = .002$). Post hoc pairwise comparisons (Bonferroni-adjusted) revealed a lower adjusted mean volume in KU group than in TU group. Specifically, the corrected mean (SE) volume of the left hippocampus was 55.46 mm^3 (SE = 1.27) in the KU group and 62.04 mm^3 (SE = 1.27) in the TU group. Confidence intervals indicated non-overlapping group estimates, supporting the robustness of the observed group difference.

Supplementary Table S5 ANCOVA Summary for Hippocampal Subfields Volume between KU participants and TU participants (Matched Sample, n = 51 per group)

Region	Group	Mean	SD	Corrected Mean	SE	95% CI Lower	95% CI Upper	F	p	partial η²	Observed Power
Left parasubiculum	KU	60.71	10.35	60.74	1.50	57.76	63.73	7.03	0.009	0.06	0.74

	TU	66.67	10.25	66.64	1.50	63.66	69.62				
Left presubiculum	KU	306.37	36.11	306.43	5.59	295.32	317.54	6.40	0.013	0.06	0.70
	TU	327.46	40.06	327.40	5.59	316.29	338.51				
Left subiculum	KU	439.28	49.93	438.60	7.44	423.83	453.37	3.94	0.050	0.03	0.50
	TU	459.79	53.13	460.47	7.44	445.70	475.24				
Left CA1	KU	615.27	68.18	611.47	9.94	591.73	631.20	5.35	0.023	0.05	0.63
	TU	641.70	74.39	645.51	9.94	625.77	665.24				
Left CA3	KU	200.72	25.39	198.57	3.49	191.63	205.51	2.11	0.149	0.02	0.30
	TU	203.95	26.23	206.09	3.49	199.15	213.04				
Left CA4	KU	244.25	23.20	242.43	3.43	235.62	249.24	4.45	0.037	0.04	0.55
	TU	251.33	26.34	253.15	3.43	246.33	259.96				
Left GC-ML-DG	KU	285.62	26.76	283.54	3.99	275.62	291.47	5.51	0.021	0.05	0.64
	TU	295.35	30.65	297.43	3.99	289.50	305.36				
Left molecular layer	KU	550.21	53.86	548.15	7.65	532.95	563.35	4.33	0.040	0.04	0.54
	TU	569.68	56.30	571.73	7.65	556.54	586.93				
**Left HATA**	**KU**	**56.33**	**8.85**	**55.46**	**1.27**	**52.93**	**57.98**	**12.27**	**<.001****	**0.11**	**0.93**
	**TU**	**61.17**	**8.72**	**62.05**	**1.27**	**59.52**	**64.57**				
Left fimbria	KU	87.42	13.86	87.37	2.54	82.32	92.42	4.70	0.032	0.04	0.57
	TU	95.49	19.82	95.53	2.54	90.49	100.58				
Left tail	KU	609.89	65.62	608.86	10.13	588.74	628.98	3.15	0.079	0.03	0.42

	TU	634.46	71.98	635.49	10.13	615.37	655.61				
Left fissure	KU	144.95	26.04	144.20	3.68	136.88	151.51	0.18	0.668	0.002	0.07
	TU	141.10	24.23	141.85	3.68	134.54	149.16				
Right parasubiculum	KU	56.33	9.48	56.01	1.34	53.34	58.67	4.24	0.042	0.04	0.53
	TU	59.78	8.74	60.10	1.34	57.44	62.77				
Right presubiculum	KU	297.92	35.05	295.75	5.18	285.47	306.04	6.19	0.015	0.05	0.69
	TU	312.67	37.92	314.84	5.18	304.55	325.12				
Right subiculum	KU	440.91	46.52	437.64	7.15	423.44	451.83	5.89	0.017	0.05	0.67
	TU	460.07	56.62	463.34	7.15	449.15	477.54				
Right CA1	KU	657.90	74.30	654.10	10.86	632.53	675.66	3.57	0.062	0.03	0.46
	TU	680.68	78.48	684.49	10.86	662.92	706.05				
Right CA3	KU	212.11	30.14	210.14	4.23	201.73	218.55	0.24	0.624	0.002	0.07
	TU	211.26	28.86	213.23	4.23	204.82	221.64				
Right CA4	KU	246.81	24.70	244.63	3.44	237.80	251.46	2.25	0.136	0.02	0.31
	TU	250.10	25.04	252.28	3.44	245.45	259.11				
Right GC ML DG	KU	290.63	27.59	288.16	4.03	280.16	296.15	2.72	0.102	0.02	0.37
	TU	295.52	30.18	297.99	4.03	290.00	305.99				
Right molecular layer	KU	569.84	55.52	566.31	7.94	550.54	582.08	4.51	0.036	0.04	0.55
	TU	587.79	58.90	591.31	7.94	575.54	607.08				

Right HATA	KU	55.44	7.97	54.79	1.16	52.49	57.10	3.782	0.055	0.03	0.48
=	TU	57.48	7.92	58.13	1.16	55.83	60.44				
Right fimbria	KU	78.35	14.64	77.53	2.47	72.62	82.44	6.114	0.015	0.05	0.68
	TU	85.76	18.66	86.58	2.47	81.67	91.49				
Right tail	KU	637.39	82.03	634.39	11.55	611.45	657.33	0.408	0.524	0.004	0.09
	TU	642.32	75.96	645.31	11.55	622.38	668.25				
Right fissure	KU	139.53	25.58	139.75	3.67	132.46	147.03	0.575	0.450	0.006	0.11
	TU	135.84	24.75	135.63	3.67	128.34	142.91				

Note. ANCOVA was conducted on a matched sample (n = 51 per group), controlling for age and years of education. Covariates in the model were evaluated at the following values: age = 22.63 years, education = 10.73 years. Pairwise comparisons were Bonferroni-corrected for multiple comparisons across 24 hippocampal subfields, with the alpha level set at .002 (0.05/24).

** Indicates Bonferroni-corrected $p < .05$ for hippocampal subfields (adjusted alpha = .002).

Post Hoc Sensitivity Analysis: Propensity Score Matching (Caliper = 0.4, n = 31 per group)

To reduce potential bias associated with baseline group differences, we conducted a post-hoc propensity score matching (PSM) analysis using more stringent criteria. A logistic regression model was employed to estimate propensity scores based on age, sex, years of education, and nicotine dependence (FTND score). Group membership served as the outcome variable (1 = ketamine use [KU]; 0 = tobacco use [TU]).

The overall model was significant (χ^2 (4) = 40.30, $p < .001$), with a Nagelkerke R^2 of .368, indicating a moderately strong prediction of group membership based on the included covariates. Among these, only years of education emerged as a statistically significant predictor (B = –0.53, SE = 0.13, Wald = 16.03, $p < .001$), suggesting that KU participants had significantly fewer years of education. Sex, age, and FTND score were not significant predictors.

Using a 1:1 nearest-neighbor matching algorithm without replacement and a caliper width of 0.2, we successfully matched 31 KU participants to 31 TU participants. Three KU participants who could not be matched were excluded. After matching, the average number of available controls matches per case was 11.44, indicating adequate match quality. In this matched sample, no significant group differences in age or years of education remained.

The matched sample demonstrated improved balance in demographic characteristics and increased classification accuracy in the logistic regression model (overall classification accuracy = 81.0%). This final matched cohort was subsequently used in all ANOVA analyses to examine group differences in bilateral hippocampal and hippocampal subfield volumes.

An analysis of variance (ANOVA) on bilateral whole hippocampal volumes

An analysis of variance (ANOVA) was conducted to compare bilateral whole hippocampal volumes between KU participants and TU participants in a matched sample (n = 31 per group).A significant group effect was observed for **the left hippocampus (F (1, 60) = 5.90, *p* = .018, partial η^2 = .09, power = .66)**, which survived Bonferroni correction for multiple comparisons (adjusted α = .025). Post hoc pairwise comparisons (Bonferroni-adjusted) revealed a lower adjusted mean volume in KU group than in TU group. Specifically, the corrected mean (SD) volume of the left hippocampus was 3482.05 mm³ (338.83) in the KU group and 3686.61 mm³ (323.74) in the TU group.

Supplementary Table S6 ANOVA Summary for Whole Hippocampal Volume between KU participants and TU participants (Matched Sample, n = 31 per group)

Region	Group	Mean	SD	SE	95% CI Lower	95% CI Upper	F	p	partial η^2	Observed Power
**Left Hippocampus**	**KU**	**3482.05**	**338.83**	**59.51**	**3363.00**	**3601.10**	**5.90**	**0.018****	**0.09**	**0.66**
	**TU**	**3686.61**	**323.74**	**59.51**	**3567.56**	**3805.66**				

Right Hippocampus	KU	3572.74	346.15	64.18	3444.35	3701.14	2.16	0.146	0.03	0.30
	TU	3706.37	368.25	64.18	3577.97	3834.76				

Note. ANCOVA was conducted on a matched sample (n = 31 per group), controlling for age and years of education. Covariates were evaluated at the following values: age = 22.63 years, education = 10.73 years. Pairwise comparisons were Bonferroni-corrected for multiple comparisons across the left and right hippocampus (2 comparisons), with the alpha level set at .025 (0.05/2).
** Indicates Bonferroni-corrected $p < .05$ for bilateral hippocampal volume comparisons (adjusted alpha = .025).

An analysis of covariance (ANCOVA) on hippocampal subfield volumes

An analysis of variance (ANOVA) was conducted to compare hippocampal subfield volumes between KU participants and TU participants in a matched sample (n = 31 per group). A significant group effect was found for the **left HATA subfield (F (1, 60) = 7.99, p = .006, partial η^2 = .11, power = .79)**; however, this effect did not remain statistically significant after Bonferroni correction for multiple comparisons (adjusted α = .002). Post hoc pairwise comparisons revealed that KU group had a significantly smaller mean volume than TU group. Specifically, the mean (SD) volume of the left HATA subfield was 55.99 mm^3 (SD = 9.46) in the KU group and 62.58 mm^3 (SD = 8.88) in the TU group.

Supplementary Table S7 ANCOVA Summary for Hippocampal Subfields Volume between KU participants and TU participants (Matched Sample, n = 31 per group)

Region	Group	Mean	SD	SE	95% CI Lower	95% CI Upper	F	p	partial η^2	Observed Power
Left parasubiculum	KU	62.04	11.09	1.97	58.09	65.99	4.30	0.042*	0.06	0.53
	TU	67.83	10.90	1.97	63.88	71.78				
Left presubiculum	KU	312.03	39.45	7.49	297.04	327.01	4.92	0.030*	0.07	0.58
	TU	335.53	43.84	7.49	320.54	350.51				
Left subiculum	KU	443.06	54.45	9.91	423.23	462.88	3.42	0.069	0.05	0.44
	TU	468.99	55.91	9.91	449.16	488.82				
Left CA1	KU	619.79	75.44	13.09	593.60	645.97	5.36	0.024*	0.08	0.62

	TU	662.65	70.24	13.09	636.46	688.84				
Left CA3	KU	202.46	25.32	4.55	193.36	211.56	1.20	0.277	0.02	0.19
	TU	209.52	25.35	4.55	200.42	218.63				
Left CA4	KU	246.54	23.08	4.37	237.79	255.30	3.16	0.080	0.05	0.41
	TU	257.55	25.58	4.37	248.79	266.30				
Left GC-ML-DG	KU	288.63	27.56	5.06	278.50	298.75	4.30	0.042*	0.06	0.53
	TU	303.48	28.79	5.06	293.36	313.61				
Left molecular layer	KU	554.64	57.62	9.95	534.72	574.56	4.51	0.038*	0.07	0.55
	TU	584.55	53.17	9.95	564.63	604.47				
**Left HATA**	**KU**	**55.98**	**9.45**	**1.64**	**52.69**	**59.28**	**7.99**	**0.006***	**0.11**	**0.79**
	**TU**	**62.57**	**8.87**	**1.64**	**59.28**	**65.87**				
Left fimbria	KU	90.17	13.53	3.24	83.69	96.65	1.88	0.175	0.03	0.27
	TU	96.47	21.63	3.24	89.99	102.95				
Left tail	KU	606.67	63.56	12.74	581.18	632.15	2.91	0.093	0.04	0.39
	TU	637.42	77.62	12.74	611.93	662.90				
Left fissure	KU	142.68	27.92	4.78	133.11	152.25	0.26	0.608	0.004	0.08
	TU	146.17	25.27	4.78	136.60	155.74				
Right parasubiculum	KU	56.79	10.00	1.71	53.36	60.22	2.21	0.142	0.03	0.31
	TU	60.41	9.074	1.71	56.98	63.84				
Right presubiculum	KU	301.18	36.00	6.93	287.30	315.06	3.11	0.083	0.04	0.41

	TU	318.51	41.10	6.93	304.63	332.39				
Right subiculum	KU	443.32	48.26	10.15	423.00	463.63	3.70	0.059	0.05	0.47
	TU	470.98	63.76	10.15	450.66	491.30				
Right CA1	KU	663.16	76.5	14.15	634.85	691.46	2.53	0.117	0.04	0.34
	TU	695.01	81.006	14.15	666.70	723.32				
Right CA3	KU	217.31	29.90	5.34	206.62	228.00	0.25	0.615	0.004	0.07
	TU	213.49	29.60	5.34	202.80	224.18				
Right CA4	KU	249.83	22.78	4.45	240.92	258.73	0.45	0.505	0.007	0.10
	TU	254.0	26.65	4.45	245.15	262.96				
Right GC ML DG	KU	294.050	25.99	5.20	283.64	304.45	0.75	0.389	0.01	0.13
	TU	300.43	31.6	5.20	290.03	310.83				
Right molecular layer	KU	575.61	55.915	10.84	553.92	597.30	2.15	0.148	0.03	0.30
	TU	598.11	64.54	10.84	576.42	619.81				
Right HATA	KU	55.37	8.76	1.49	52.39	58.35	1.16	0.286	0.01	0.18
=	TU	57.64	7.81	1.49	54.66	60.62				
Right fimbria	KU	78.40	16.54	3.21	71.98	84.82	3.42	0.069	0.05	0.44
	TU	86.80	19.12	3.21	80.38	93.22				
Right tail	KU	637.69	90.50	15.61	606.47	668.92	0.35	0.552	0.006	0.09
	TU	650.89	83.16	15.61	619.67	682.11				
Right fissure	KU	142.55	27.84	5.05	132.45	152.65	0.14	0.710	0.002	0.06

	TU	139.88	28.38	5.05	129.78	149.98			

Note. ANCOVA was conducted on a matched sample (n = 31 per group), controlling for age and years of education. Covariates were evaluated at the following values: age = 22.63 years, education = 10.73 years. Pairwise comparisons were Bonferroni-corrected for multiple comparisons across 24 hippocampal subfields, with the alpha level set at .002 (0.05/24).

***** Indicates un-corrected $p < .05$ for hippocampal subfields.

Post Hoc Sensitivity Analysis: Controlling for Psychiatric Symptoms (GSI), Group × Sex Interaction, and Group × FTND Interaction

An analysis of covariance (ANCOVA) on bilateral whole hippocampal volumes

An analysis of covariance (ANCOVA) was conducted to compare bilateral whole hippocampal volumes between KU participants and TU participants , controlling for age, sex, years of education, nicotine dependence (FTND), and **general psychiatric symptoms (GSI)**.

A significant main effect of group was found for **the left hippocampus**, **F (1, 92) = 6.89, p = .010, partial η^2 = .07, observed power = .73.** A group effect was also observed for the right hippocampus**,** F (1, 92) = 4.52, p = .036, partial η^2 = .04, observed power = .55. However, only the left hippocampal effect remained significant after Bonferroni correction for multiple comparisons (adjusted α = .025). Post hoc pairwise comparisons (Bonferroni-adjusted) revealed a lower adjusted mean volume in KU group than in TU group.

No significant Group × Sex interaction was found for either hemisphere (left F (1, 92) = 0.04, p = .838; right: F (1, 92) = 0.2, p = .654), indicating that sex did not moderate the group differences in hippocampal volume.

A significant Group × FTND interaction was observed for the left hippocampus, F (1, 92) = 7.69, p = .007, partial η^2 = .07, observed power = .78, suggesting that the relationship between nicotine dependence and hippocampal volume differed between groups. Specifically, the association between FTND scores and hippocampal volume may be negative in KU participants and non-significant or in the opposite direction in TU participants. A marginal interaction was also found for the right hippocampus, F (1, 92) = 5.27, p = .024, partial η^2 = .05, observed power = .62; however, this did not survive Bonferroni correction (adjusted α = .025).

The Group × GSI interaction was not significant for either region (left F (1, 92) = 0.73, p = .395; right: F (1, 92) = 0.45, p = .503), indicating that general psychiatric symptoms did not significantly moderate the observed group differences.

Supplementary Table S8 ANOVA Summary for Whole Hippocampal Volume between KU participants and TU participants (Psychiatric Symptoms)

	group	Mean	SD	Mean (corrected)	SE	95% Confidence Interval		F	Sig.	partial η²	Observed Power	F (Group × Sex)	p (Group × Sex)	F (Group ×FTND)	p (Group × FTND)	F (Group × GSI)	p (Group × GSI)
**Left Hippocampus**	**KU**	**3456.11**	**316.34**	**3429.84**	**42.85**	**3344.72**	**3514.96**	**6.97**	**0.01****	**0.0**	**0.74**	**0.04**	**0.838**	**7.68**	**0.007****	**0.73**	**0.395**
	**TU**	**3607.09**	**332.44**	**3595.81**	**42.6**	**3511.08**	**3680.55**										

Right Hippocampus	KU	3543.69	321.70	3509.75	43.89	3422.57	3596.93	4.49	0.03	0.04	0.55	0.20	0.654	5.26	0.024	0.45	0.503
	TU	3643.46	341.48	3646.20	43.69	3559.41	3732.98										
** Indicates Bonferroni-corrected *p* < .05 for bilateral hippocampal volume comparisons (adjusted alpha = .025).																	

An analysis of covariance (ANCOVA) on hippocampal subfield volumes

An analysis of covariance (ANCOVA) was conducted to compare hippocampal subfield volumes between KU participants and TU participants , controlling for age, sex, years of education, nicotine dependence (FTND), and **general psychiatric symptoms (GSI).**

A significant main effect of group was found for the left HATA, F (1, 92) = 13.10, p < .001, partial η^2 = .125, observed power = .948. This effect remained significant after Bonferroni correction for multiple comparisons (adjusted α = .002). Post hoc pairwise comparisons (Bonferroni-adjusted) revealed a lower adjusted mean volume in KU group than in TU group.

No significant **Group × Sex** interaction was found for the left HATA, F (1, 92) = 0.02, p = .887, indicating that sex did not moderate the group differences in HATA volume.

No significant **Group × FTND** interaction was observed for the left HATA, F (1, 92) = 1.35, p = .247, suggesting that nicotine dependence did not moderate the group effect.

No significant **Group × GSI** interaction was found for the left HATA, F (1, 92) = 1.18, p = .281, indicating that general psychiatric symptoms did not significantly influence the group difference.

Supplementary Table S9 ANCOVA Summary for Hippocampal Subfields Volume between KU participants and TU participants (Psychiatric Symptoms)

	group	Mean	SD	Mean (corrected)	SE	95% Confidence Interval		F	Sig.	partial η²	Observed Power	F (Group × Sex)	p (Group × Sex)	F (Group ×FTND)	p (Group × FTND)	F (Group × GSI)	p (Group × GSI)
Left parasubiculum	KU	60.71	10.35	60.90	1.54	57.84	63.96	5.99	0.016	0.06	0.68	0.05	0.824	0.05	0.830	0.04	0.836
	TU	66.67	10.25	66.43	1.53	63.38	69.47										
Left presubiculum	KU	306.37	36.11	305.56	5.54	294.54	316.57	5.81	0.018	0.06	0.66	0.05	0.829	3.19	0.077	0.23	0.636
	TU	327.46	40.06	325.14	5.52	314.18	336.11										
Left subiculum	KU	439.28	49.93	437.57	7.37	422.92	452.23	3.17	0.079	0.03	0.42	0.26	0.610	4.96	0.028	0.10	0.750
	TU	459.79	53.13	456.81	7.34	442.23	471.40										
Left CA1	KU	615.27	68.18	609.12	9.40	590.44	627.81	5.09	0.026	0.05	0.61	0.80	0.373	6.02	0.016	1.26	0.265
	TU	641.70	74.39	640.25	9.36	621.65	658.85										
Left CA3	KU	200.71	25.39	198.42	3.42	191.62	205.22	2.06	0.154	0.02	0.30	0.06	0.806	0.41	0.522	0.01	0.917
	TU	203.95	26.23	205.63	3.40	198.86	212.40										
Left CA4	KU	244.25	23.20	241.95	3.31	235.38	248.53	4.15	0.045	0.04	0.52	0.16	0.689	2.85	0.095	0.38	0.539
	TU	251.33	26.34	251.84	3.29	245.30	258.39										
Left GC-ML-DG	KU	285.62	26.76	283.08	3.83	275.47	290.70	5.16	0.025	0.05	0.61	0.21	0.648	2.59	0.111	0.52	0.474
	TU	295.35	30.65	295.85	3.81	288.28	303.44										
Left molecular layer	KU	550.21	53.86	546.49	7.30	531.99	561.01	3.99	0.049	0.04	0.51	0.26	0.613	5.83	0.018	0.59	0.444
	TU	569.68	56.30	567.87	7.27	553.43	582.32										

**Left HATA**	**KU**	**56.33**	**8.85**	**55.27**	**1.20**	**52.88**	**57.66**	**13.10**	**<.001**	**0.13**	**0.95**	**0.02**	**0.887**	**1.36**	**0.247**	**1.18**	**0.281**
	**TU**	**61.17**	**8.72**	**61.65**	**1.19**	**59.28**	**64.03**										
Left fimbria	KU	87.42	13.86	87.14	2.48	82.22	92.07	3.37	0.070	0.04	0.44	0.49	0.487	4.91	0.029	1.82	0.181
	TU	95.48	19.82	93.82	2.46	88.92	98.72										
Left tail	KU	609.89	65.62	604.29	9.88	584.66	623.93	3.27	0.074	0.03	0.43	1.25	0.266	9.57	0.003	0.37	0.547
	TU	634.45	71.98	630.48	9.80	610.94	650.03										
Left fissure	KU	144.95	26.04	143.50	3.65	136.25	150.76	0.12	0.727	0.00	0.06	0.36	0.553	1.69	0.197	0.27	0.608
	TU	141.10	24.23	141.62	3.63	134.40	148.85										
Right parasubiculum	KU	56.33	9.48	56.17	1.40	53.40	58.96	3.44	0.067	0.04	0.45	0.10	0.749	0.03	0.870	0.00	0.968
	TU	59.78	8.74	59.98	1.39	57.21	62.75										
Right presubiculum	KU	297.92	35.05	294.53	5.19	284.22	304.85	5.77	0.018	0.06	0.66	0.18	0.671	2.31	0.132	1.67	0.199
	TU	312.67	37.92	312.82	5.16	302.56	323.09										
Right subiculum	KU	440.91	46.52	435.53	7.04	421.53	449.53	5.64	0.020	0.06	0.65	0.60	0.440	5.42	0.022	1.07	0.304
	TU	460.07	56.61	460.07	7.01	446.14	474.01										
Right CA1	KU	657.90	74.30	651.97	10.40	631.18	672.77	3.18	0.078	0.03	0.42	0.51	0.477	5.05	0.027	0.52	0.475
	TU	680.68	78.48	679.33	10.42	658.63	700.03										
Right CA3	KU	212.11	30.14	211.05	4.21	202.69	219.42	0.12	0.734	0.00	0.06	0.19	0.662	0.00	0.985	1.34	0.250
	TU	211.26	28.86	213.15	4.19	204.83	221.48										
Right CA4	KU	246.81	24.70	244.73	3.34	238.11	251.37	1.85	0.177	0.02	0.27	0.09	0.771	1.60	0.209	0.43	0.515

	TU	250.10	25.04	251.40	3.32	244.80	258.00										
Right GC ML DG	KU	290.63	27.59	288.35	3.85	280.69	296.01	2.26	0.136	0.02	0.32	0.15	0.703	1.86	0.176	0.51	0.478
	TU	295.52	30.17	296.85	3.84	289.23	304.48										
Right molecular layer	KU	569.84	55.51	565.01	7.71	549.70	580.33	4.16	0.044	0.04	0.52	0.13	0.722	4.04	0.047	0.14	0.711
	TU	587.79	58.90	588.06	7.67	572.82	603.31										
Right HATA	KU	55.44	7.97	54.87	1.17	52.55	57.21	2.99	0.087	0.03	0.40	0.28	0.600	0.61	0.437	0.01	0.924
=	TU	57.48	7.92	57.84	1.16	55.50	60.17										
Right fimbria	KU	78.35	14.64	77.08	2.43	72.24	81.92	4.92	0.029	0.05	0.59	0.09	0.772	4.72	0.032	2.84	0.096
	TU	85.76	18.66	84.99	2.42	80.18	89.82										
Right tail	KU	637.39	82.03	630.41	11.27	608.02	652.81	0.46	0.497	0.01	0.10	0.01	0.926	3.83	0.053	0.94	0.335
	TU	642.32	75.96	641.67	11.22	619.38	663.97										
Right fissure	KU	139.53	25.58	138.98	3.62	131.79	146.17	0.66	0.419	0.01	0.13	0.29	0.593	1.83	0.180	0.72	0.399
	TU	135.84	24.75	134.67	3.60	127.51	141.83										
** Indicates Bonferroni-corrected p < .05 for hippocampal subfields (adjusted alpha = .002).																	

Post Hoc Sensitivity Analyses for Confounding Control: Ketamine use intensity

To evaluate whether the observed group differences in hippocampal volume could be attributed to variability in ketamine use intensity, we conducted partial correlation analyses within the KU group ($n = 51$). Specifically, we examined the associations between hippocampal volumes and ketamine use frequency (days per month) as well as duration of use (in years), controlling for age, sex, years of education, nicotine dependence (FTND score), and general psychiatric symptom severity (GSI score).

The results indicated no significant associations between ketamine use intensity and hippocampal volume. For the left whole hippocampus, neither frequency ($r = .07$, $p = .626$) nor duration ($r = .09$, $p = .542$) was significantly correlated with volume. Similarly, for the left hippocampal–amygdaloid transition area (HATA), correlations with frequency ($r = –.09$, $p = .555$) and duration ($r = –.07$, $p = .631$) were nonsignificant.

Post Hoc Sensitivity Analysis: Controlling for Nicotine-Related Variables

An analysis of covariance (ANCOVA) on bilateral whole hippocampal volumes

An analysis of covariance (ANCOVA) was conducted to compare bilateral whole hippocampal volumes between KU participants and TU participants, controlling for age, sex, years of education, nicotine dependence (FTND), TIV, **daily cigarette consumption and tobacco onset age**.

A significant main effect of group was found for **the left hippocampus, F (1, 117) = 6.73, p = .01, partial η^2 = .05, observed power = .73**. A group effect was no observed for the right hippocampus, F (1, 117) = 3.02, p = .085, partial η^2 = .03, observed power = .41. The left hippocampal effect remained significant after Bonferroni correction for multiple comparisons (adjusted α = .025). Post hoc pairwise comparisons (Bonferroni-adjusted) revealed a lower adjusted mean volume in KU group than in TU group.

Supplementary Table S10 ANOVA Summary for Whole Hippocampal Volume between KU participants and TU participants (Nicotine-Related Variables)

	group	Mean	Std. Deviation	Mean (corrected)	Std. Error	95% Confidence Interval		F	Sig.	Partial Eta Squared	Observed Power
**Left Hippocampus**	**KU**	**3451.65**	**308.52**	**3443.064[a]**	**36.27**	**3371.24**	**3514.89**	**6.73**	**0.011****	**0.05**	**0.73**
	**TU**	**3570.13**	**324.63**	**3576.567[a]**	**30.61**	**3515.94**	**3637.19**				
Right Hippocampus	KU	3535.67	315.80	3521.776[a]	38.48	3445.56	3597.99	3.02	0.085	0.03	0.41
	TU	3606.20	331.91	3616.624[a]	32.48	3552.29	3680.96				

** Indicates Bonferroni-corrected $p < .05$ for bilateral hippocampal volume comparisons (adjusted alpha = .025).

An analysis of covariance (ANCOVA) on hippocampal subfield volumes

An analysis of covariance (ANCOVA) was conducted to compare bilateral whole hippocampal volumes between KU participants and TU participants, controlling for age, sex, years of education, nicotine dependence (FTND), TIV, daily cigarette consumption and tobacco onset age.

A significant main effect of group was found for the left HATA, *F* (1, 117) = 12.38, $p < .001$, partial $\eta^2 = .09$, observed power = .94. This effect remained significant after Bonferroni correction for multiple comparisons (adjusted $\alpha = .002$). Post hoc pairwise comparisons (Bonferroni-adjusted) revealed a lower adjusted mean volume in KU group than in TU group.

Supplementary Table S11 ANCOVA Summary for Hippocampal Subfields Volume between KU participants and TU participants (Nicotine-Related Variables)

Region	Group	Mean	SD	Mean (corrected)	SE	95% CI Lower	95% CI Upper	F	p	partial η²	Observed Power
Left parasubiculum	KU	60.28	10.37	60.78	1.43	57.94	63.61	4.29	0.041	0.04	0.54
	TU	65.36	9.67	64.99	1.21	62.59	67.38				
Left presubiculum	KU	304.37	36.12	307.31	5.03	297.34	317.27	4.08	0.046	0.03	0.52
	TU	323.92	39.48	321.72	4.25	313.31	330.13				
Left subiculum	KU	438.19	48.86	440.79	6.47	427.97	453.61	2.84	0.094	0.02	0.39
	TU	458.23	53.60	456.28	5.47	445.46	467.10				
Left CA1	KU	616.63	66.64	611.83	8.36	595.27	628.39	4.46	0.037	0.04	0.55
	TU	633.30	74.53	636.90	7.06	622.92	650.88				
Left CA3	KU	201.05	24.72	197.28	3.17	191.01	203.55	2.21	0.140	0.02	0.31
	TU	201.12	24.71	203.95	2.67	198.66	209.24				
Left CA4	KU	243.83	22.74	242.30	2.75	236.86	247.75	3.66	0.058	0.03	0.48

	TU	248.62	23.69	249.76	2.32	245.16	254.35				
Left GC-ML-DG	KU	285.23	26.10	283.58	3.12	277.39	289.76	5.08	0.026	0.04	0.61
	TU	292.31	27.99	293.55	2.64	288.34	298.77				
Left molecular layer	KU	549.81	52.40	548.73	6.28	536.30	561.16	3.49	0.064	0.03	0.46
	TU	564.55	55.07	565.37	5.30	554.87	575.86				
**Left HATA**	**KU**	**56.54**	**8.85**	**55.37**	**1.12**	**53.16**	**57.58**	**12.38**	**<.001****	**0.10**	**0.94**
	**TU**	**60.06**	**8.20**	**60.94**	**0.94**	**59.07**	**62.81**				
Left fimbria	KU	87.42	13.74	88.90	2.21	84.52	93.29	2.26	0.135	0.02	0.32
	TU	94.74	18.54	93.63	1.87	89.93	97.33				
Left tail	KU	608.31	65.45	606.20	10.44	585.52	626.88	2.47	0.119	0.02	0.35
	TU	627.91	75.58	629.49	8.82	612.03	646.95				
Left fissure	KU	144.30	25.72	143.51	3.57	136.45	150.57	0.07	0.792	0.00	0.06
	TU	141.58	24.20	142.17	3.01	136.21	148.13				
Right parasubiculum	KU	55.91	9.46	56.13	1.37	53.42	58.83	3.81	0.053	0.03	0.49
	TU	60.08	8.62	59.91	1.15	57.63	62.20				
Right presubiculum	KU	296.17	34.85	295.46	4.68	286.19	304.74	6.20	0.014	0.05	0.69

	TU	311.47	38.19	312.00	3.95	304.17	319.83				
Right subiculum	KU	438.84	46.39	437.09	6.19	424.84	449.34	6.06	0.015	0.05	0.69
	TU	457.38	53.79	458.69	5.22	448.35	469.03				
Right CA1	KU	657.78	72.22	654.29	10.01	634.45	674.12	1.50	0.223	0.01	0.23
	TU	669.08	79.33	671.70	8.45	654.96	688.44				
Right CA3	KU	212.20	29.37	210.56	3.97	202.70	218.41	0.13	0.725	0.00	0.06
	TU	207.34	27.90	208.57	3.35	201.94	215.20				
Right CA4	KU	246.08	24.19	244.67	3.03	238.68	250.66	0.53	0.469	0.00	0.11
	TU	246.72	23.71	247.79	2.55	242.73	252.84				
Right GC ML DG	KU	290.08	26.91	288.68	3.49	281.77	295.59	0.76	0.385	0.01	0.14
	TU	291.95	28.80	293.00	2.95	287.16	298.83				
Right molecular layer	KU	568.85	54.13	566.70	7.01	552.81	580.58	2.45	0.120	0.02	0.34
	TU	580.65	58.14	582.26	5.92	570.54	593.99				
Right HATA	KU	55.72	7.85	55.20	1.13	52.96	57.45	2.29	0.133	0.02	0.32
	TU	57.24	8.16	57.63	0.96	55.74	59.52				
Right fimbria	KU	77.85	14.45	77.81	2.27	73.31	82.32	6.03	0.016	0.05	0.68
	TU	85.71	17.70	85.74	1.92	81.93	89.54				

Right tail	KU	636.21	84.02	635.19	11.30	612.82	657.57	0.07	0.797	0.00	0.06
	TU	638.57	75.27	639.33	9.54	620.44	658.22				
Right fissure	KU	139.32	25.66	139.35	3.45	132.52	146.18	0.78	0.379	0.01	0.14
	TU	135.05	23.65	135.03	2.91	129.26	140.80				
** Indicates Bonferroni-corrected $p < .05$ for hippocampal subfields (adjusted alpha = .002).											

Post Hoc Sensitivity Analyses for Confounding Control: Tobacco Use Intensity

To evaluate whether group-level differences in hippocampal volume could be attributed to variability in tobacco use intensity, we conducted partial correlation analyses within both the KU participants and tobacco use control (TU) groups. Specifically, we assessed the associations between hippocampal volumes and two indices of tobacco use—frequency (days per week) and duration (in years)—while controlling for age, sex, years of education, nicotine dependence (FTND score), and total intracranial volume (TIV).

For the **left hippocampus**, correlation coefficients for frequency and duration of tobacco use were as follows:

- KU group: frequency, $r = -0.13$, $p = .386$; duration, $r = -0.06$, $p = .706$
- TU group: frequency, $r = 0.03$, $p = .810$; duration, $r = 0.16$, $p = .199$

For the **left hippocampal-amygdaloid transition area (HATA)**:

- KU group: frequency, $r = -0.22$, $p = .118$; duration, $r = -0.11$, $p = .467$
- TU group: frequency, $r = -0.11$, $p = .373$; duration, $r = 0.07$, $p = .558$

None of these correlations reached statistical significance. These findings suggest that the observed group-level reductions in hippocampal and subfield (HATA) volumes are unlikely to be driven by variability in tobacco use frequency or duration.

Post Hoc Sensitivity Analysis: Controlling for Broader Substance Use (ACOMP and DCOMP)

An analysis of covariance (ANCOVA) on bilateral whole hippocampal volumes

An analysis of covariance (ANCOVA) was conducted to compare bilateral whole hippocampal volumes between KU participants and TU participants, controlling for age, sex, years of education, nicotine dependence (FTND), TIV, **ACOMP and DCOMP**.

A significant main effect of group was found for **the left hippocampus, F (1, 117) = 5.83, p = .017, partial η^2 = .05, observed power = .67**. A group effect was no observed for the right hippocampus, F (1, 117) = 2.35, p = .0128, partial η^2 = .02, observed power = .33. The left hippocampal effect remained significant after Bonferroni correction for multiple comparisons (adjusted α = .025). Post hoc pairwise comparisons (Bonferroni-adjusted) revealed a lower adjusted mean volume in KU group than in TU group.

Supplementary Table S12 ANOVA Summary for Whole Hippocampal Volume between KU participants and TU participants (Broader Substance Use)

	Group	Mean	Std. Deviation	Mean (corrected)	Std. Error	95% Confidence Interval		F	Sig.	partial η^2	Observed Power
**Left Hippocampus**	**KU**	**3451.65**	**308.52**	**3438.60**	**39.92**	**3359.55**	**3517.65**	**5.83**	**0.017****	**0.05**	**0.67**
	**TU**	**3570.13**	**324.63**	**3579.92**	**33.22**	**3514.12**	**3645.71**				
Right Hippocampus	KU	3535.67	315.80	3521.96	42.08	3438.62	3605.29	2.35	0.128	0.02	0.33
	TU	3606.20	331.91	3616.49	35.02	3547.13	3685.85				

** Indicates Bonferroni-corrected $p < .05$ for bilateral hippocampal volume comparisons (adjusted alpha = .025).

An analysis of covariance (ANCOVA) on hippocampal subfield volumes

An analysis of covariance (ANCOVA) was conducted to compare bilateral whole hippocampal volumes between KU participants and TU participants, controlling for age, sex, years of education, nicotine dependence (FTND), TIV, **ACOMP and DCOMP**.

A significant main effect of group was found for the left HATA, F (1, 117) = 11.05, p = .001, partial η^2 = .09, observed power = .91. This effect remained significant after Bonferroni correction for multiple comparisons (adjusted α = .002). Post hoc pairwise comparisons (Bonferroni-adjusted) revealed a lower adjusted mean volume in KU group than in TU group.

Supplementary Table S13 ANCOVA Summary for Hippocampal Subfields Volume between KU participants and TU participants (Broader Substance Use)

Region	Group	Mean	SD	Mean (corrected)	SE	95% CI Lower	95% CI Upper	F	p	partial η^2	Observed Power
Left parasubiculum	KU	60.27	10.37	60.67	1.56	57.58	63.75	3.71	0.056	0.03	0.48
	TU	65.36	9.67	65.07	1.30	62.50	67.64				
Left presubiculum	KU	304.37	36.12	305.32	5.37	294.68	315.96	5.16	0.025	0.04	0.62
	TU	323.92	39.48	323.21	4.47	314.35	332.06				
Left subiculum	KU	438.19	48.86	437.99	6.97	424.18	451.81	3.97	0.049	0.03	0.51
	TU	458.23	53.60	458.38	5.81	446.88	469.87				
Left CA1	KU	616.62	66.64	610.97	9.32	592.51	629.42	3.78	0.054	0.03	0.49
	TU	633.30	74.53	637.55	7.76	622.18	652.91				
Left CA3	KU	201.05	24.72	198.32	3.52	191.35	205.29	0.88	0.349	0.01	0.15
	TU	201.12	24.70	203.17	2.93	197.37	208.97				
Left CA4	KU	243.83	22.74	242.23	3.03	236.23	248.24	2.90	0.091	0.02	0.39

	TU	248.62	23.68	249.81	2.52	244.81	254.81				
Left GC-ML-DG	KU	285.23	26.10	283.78	3.45	276.95	290.62	3.60	0.060	0.03	0.47
	TU	292.31	27.99	293.40	2.87	287.71	299.09				
Left molecular layer	KU	549.81	52.40	547.55	6.94	533.81	561.30	3.37	0.069	0.03	0.45
	TU	564.55	55.07	566.25	5.78	554.81	577.68				
**Left HATA**	**KU**	**56.54**	**8.85**	**55.19**	**1.21**	**52.80**	**57.58**	**11.05**	**0.001****	**0.09**	**0.91**
	**TU**	**60.06**	**8.20**	**61.08**	**1.01**	**59.09**	**63.07**				
Left fimbria	KU	87.42	13.74	89.09	2.41	84.32	93.86	1.55	0.216	0.01	0.23
	TU	94.74	18.54	93.49	2.01	89.52	97.46				
Left tail	KU	608.31	65.45	607.49	11.21	585.29	629.68	1.64	0.203	0.01	0.25
	TU	627.91	75.58	628.53	9.33	610.05	647.00				
Left fissure	KU	144.30	25.72	143.69	3.85	136.06	151.31	0.09	0.771	0.00	0.06
	TU	141.58	24.20	142.04	3.20	135.70	148.39				
Right parasubiculum	KU	55.90	9.46	57.06	1.44	54.21	59.91	1.04	0.310	0.01	0.17
	TU	60.08	8.62	59.21	1.20	56.84	61.58				
Right presubiculum	KU	296.17	34.85	296.63	5.11	286.52	306.75	3.74	0.055	0.03	0.48

	TU	311.47	38.19	311.12	4.25	302.71	319.54				
Right subiculum	KU	438.84	46.39	435.85	6.80	422.39	449.31	5.69	0.019	0.05	0.66
	TU	457.38	53.79	459.63	5.66	448.42	470.83				
Right CA1	KU	657.78	72.22	652.95	10.94	631.29	674.61	1.52	0.221	0.01	0.23
	TU	669.08	79.33	672.70	9.10	654.68	690.73				
Right CA3	KU	212.20	29.37	211.75	4.25	203.34	220.17	0.43	0.514	0.00	0.10
	TU	207.34	27.90	207.68	3.54	200.67	214.68				
Right CA4	KU	246.08	24.19	245.37	3.30	238.84	251.90	0.15	0.697	0.00	0.07
	TU	246.72	23.71	247.26	2.75	241.82	252.69				
Right GC ML DG	KU	290.08	26.91	289.69	3.81	282.15	297.23	0.21	0.648	0.00	0.07
	TU	291.95	28.80	292.24	3.17	285.97	298.52				
Right molecular layer	KU	568.85	54.13	566.00	7.65	550.85	581.16	2.23	0.138	0.02	0.32
	TU	580.65	58.14	582.78	6.37	570.17	595.40				
Right HATA	KU	55.72	7.84	55.32	1.23	52.89	57.74	1.54	0.217	0.01	0.23
	TU	57.24	8.16	57.55	1.02	55.53	59.56				
Right fimbria	KU	77.85	14.45	78.40	2.49	73.46	83.34	3.56	0.062	0.03	0.46
	TU	85.71	17.70	85.30	2.08	81.19	89.41				

Right tail	KU	636.21	84.02	632.94	12.06	609.06	656.81	0.21	0.648	0.00	0.07
	TU	638.57	75.27	641.02	10.04	621.15	660.90				
Right fissure	KU	139.32	25.66	139.56	3.74	132.16	146.97	0.73	0.394	0.01	0.14
	TU	135.05	23.65	134.87	3.11	128.71	141.04				

** Indicates Bonferroni-corrected p < .05 for hippocampal subfields (adjusted alpha = .002).
